# Measuring acoustic habitats

**DOI:** 10.1111/2041-210X.12330

**Published:** 2015-01-27

**Authors:** Nathan D Merchant, Kurt M Fristrup, Mark P Johnson, Peter L Tyack, Matthew J Witt, Philippe Blondel, Susan E Parks

**Affiliations:** 1 Centre for Environment, Fisheries & Aquaculture Science (Cefas)Lowestoft, Suffolk, NR33 0HT, UK; 2 Department of Biology, Syracuse UniversitySyracuse, NY, 13244, USA; 3 Department of Physics, University of BathBath, BA2 7AY, UK; 4 Natural Sounds and Night Skies Division, National Park ServiceFort Collins, CO, 80525, USA; 5 Scottish Oceans Institute, University of St. AndrewsSt. Andrews, Fife, KY16 8LB, UK; 6 Environment and Sustainability Institute, University of ExeterPenryn, TR10 9FE, UK

**Keywords:** acoustic ecology, ambient noise, anthropogenic noise, bioacoustics, ecoacoustics, habitat monitoring, passive acoustic monitoring, remote sensing, soundscape

## Abstract

**1.** Many organisms depend on sound for communication, predator/prey detection and navigation. The acoustic environment can therefore play an important role in ecosystem dynamics and evolution. A growing number of studies are documenting acoustic habitats and their influences on animal development, behaviour, physiology and spatial ecology, which has led to increasing demand for passive acoustic monitoring (PAM) expertise in the life sciences. However, as yet, there has been no synthesis of data processing methods for acoustic habitat monitoring, which presents an unnecessary obstacle to would-be PAM analysts.

**2.** Here, we review the signal processing techniques needed to produce calibrated measurements of terrestrial and aquatic acoustic habitats. We include a supplemental tutorial and template computer codes in matlab and r, which give detailed guidance on how to produce calibrated spectrograms and statistical analyses of sound levels. Key metrics and terminology for the characterisation of biotic, abiotic and anthropogenic sound are covered, and their application to relevant monitoring scenarios is illustrated through example data sets. To inform study design and hardware selection, we also include an up-to-date overview of terrestrial and aquatic PAM instruments.

**3.** Monitoring of acoustic habitats at large spatiotemporal scales is becoming possible through recent advances in PAM technology. This will enhance our understanding of the role of sound in the spatial ecology of acoustically sensitive species and inform spatial planning to mitigate the rising influence of anthropogenic noise in these ecosystems. As we demonstrate in this work, progress in these areas will depend upon the application of consistent and appropriate PAM methodologies.

## Introduction

The increasing sophistication of passive acoustic monitoring (PAM) – the recording of sound in a habitat – has led to new insights in the study of acoustically sensitive organisms over a wide range of spatial and temporal scales (Van Parijs *et al*. 2009; Blumstein *et al*. 2011). Such studies point towards the fundamental role of sound for many species and ecosystems, mediating processes as diverse as predator–prey interactions (Remage-Healey, Nowacek & Bass 2006), larval settlement (Simpson *et al*. 2005) and coordinated behaviour (Boinski & Campbell 1995).

The acoustical backdrop to these phenomena is by no means a silent world: the evolution of acoustic signalling has taken place in the context of a varying natural background (Wiley & Richards 1978; Brumm & Slabbekoorn 2005) to which organisms adapt their acoustic behaviour (Morton 1975). This background sound is generated by weather processes (wind, rain, thunder), seismic events and competing biotic sound (Hildebrand 2009; Pijanowski *et al*. 2011). However, since the advent of large-scale industrialisation, acoustic habitats have become increasingly disrupted by anthropogenic noise. On land, these sources include road, rail and air transport, and industrial activity (Barber, Crooks & Fristrup 2010), while underwater, shipping, offshore construction, oil and gas exploration, and sonar operations contribute to the soundscape (Payne & Webb 1971; Hildebrand 2009). In both domains, this noise can mask acoustic cues (Brumm & Slabbekoorn 2005; Clark *et al*. 2009) and elicit behavioural responses (Sun & Narins 2005; Nowacek *et al*. 2007), with the potential to cause chronic physiological stress (Rolland *et al*. 2012; Francis & Barber 2013) and wider effects on populations (National Research Council 2005) and communities (Francis, Ortega & Cruz 2009). Awareness of these human impacts has brought renewed urgency to the study of acoustic habitats and their influences on ecosystem processes (Francis & Barber 2013).

The rapid expansion of this field has been driven by major advances in PAM technology and has led to growing demand for acoustics expertise in the life sciences. Over the past two decades, the development of cost-effective autonomous recording units has revolutionised bioacoustics in both air (Mennill *et al*. 2012; Digby *et al*. 2013) and underwater (Sousa-Lima *et al*. 2013). Marine bioacoustics has been particularly driven by technological innovation, with new perspectives offered by non-invasive recording tags (Burgess *et al*. 1998; Johnson & Tyack 2003), autonomous gliders (Rudnick, Davis & Eriksen 2004; Baumgartner & Fratantoni 2008), drifting platforms (Wilson, Benjamins & Elliott 2013) and region-scale cabled ocean observatories (Favali, Beranzoli & de Santis 2015).

However, there is currently a lack of clear guidance on how to analyse PAM data to produce calibrated measurements. Calibrated data provide absolute measures of biotic, abiotic and anthropogenic sound levels, which are necessary to draw meaningful comparisons of habitats through time and at different locations. Here, we seek to address this deficit through a user-friendly guide to the methods that underpin the study of acoustic habitats. We detail the signal processing steps required to produce absolute measurements of sound pressure and demonstrate the use of analytical techniques to describe variability and trends in sound levels and to characterise discrete acoustic events. In doing so, we emphasise the considerable overlap in acoustic analysis methods in air and underwater, and the potential for both terrestrial and aquatic bioacoustics to benefit from greater integration and knowledge exchange.

## Monitoring platforms

Technology has shaped the development of bioacoustics. Advances in instrumentation, data storage capacity and data analysis capabilities have opened up new avenues of research while enriching established areas. To help contextualise historical constraints on data collection and inform the design of future habitat monitoring programs, this section briefly summarises the capabilities and limitations of the main types of PAM platform. We first consider *fixed platforms* – those designed to be deployed at one location for days or longer – and then *mobile platforms* – those that record while in motion or are portable and deployed for short periods.

### Fixed platforms

The recent expansion in the study of acoustic habitats has been facilitated by the development of autonomous acoustic recorders: self-contained digital instruments that can be fixed to terrestrial structures or moored to the seafloor to record the soundscape continuously on the scale of months (Mennill *et al*. 2012; Sousa-Lima *et al*. 2013; see Table 1 for a selection of commercially available devices). These are generally more cost-effective and easier to deploy than cabled systems and can be positioned in arrays to investigate spatial characteristics of acoustic habitats and to localise and track sound sources (Van Parijs *et al*. 2009; Blumstein *et al*. 2011). Each unit consists of battery-powered electronics for digital data acquisition and storage within a weather- or waterproof housing. The acoustic transducer (microphone or hydrophone) may be mounted on the device or attached via a cable. Deployment longevity is limited by power consumption and data storage capacity, both of which continue to improve as the technology evolves (Sousa-Lima *et al*. 2013). Supplementing the inbuilt power supply with an external source (solar panel or additional battery) or duty cycling the ‘on time’ can increase longevity. Habitat monitoring can continue indefinitely if recorders are regularly serviced to replenish batteries and data storage, and systems are also being developed for remote data retrieval over wireless communication networks (e.g. Wildlife Acoustics Song Stream; SMRU Marine PAMBuoy). Seafloor-mounted systems can be recovered using acoustic release devices, although acoustic release malfunction and trawling by fishing vessels are common field hazards (Dudzinski *et al*. 2011). Theft and vandalism are also potential concerns (Clarin *et al*. 2013), as well as damage from wildlife and biofouling.

**Table 1:** Selection of commercially available integrated acoustic habitat recorders

Manufacturer	Model	Channels	Maximum sampling rate (kHz)	Domain
Wildlife Acoustics	SM2+	2	96	Air
Wildlife Acoustics	SM3	2	96	Air
Jasco Applied Sciences	AMAR G3	9	687·5	Underwater
Loggerhead Instruments	DSG-ST	1	288	Underwater
Ocean Instruments	SoundTrap 202HF	1	576	Underwater
Wildlife Acoustics	SM3M	2	192	Underwater

Cabled systems have long been used to monitor underwater sound (Wenz 1961) and consist of seafloor-mounted hydrophones connected to shore stations providing power and data acquisition. In recent years, cable-mounted systems have seen a resurgence with the expansion of cabled ocean observatories, for example the NEPTUNE, VENUS and RSN networks in the North-east Pacific (Favali, Beranzoli & de Santis 2015). The capacity for real-time acoustic monitoring and the less frequent servicing required (compared to autonomous units) are the principal advantages of cabled systems, although the associated costs of deployment, maintenance and management of these long-term devices and the large volumes of data they generate are correspondingly high.

### Mobile platforms

In air, a convenient and portable tool for acoustic habitat monitoring is the commercial sound level meter, which makes calibrated measurements of sound pressure level (SPL; Table 2; Appendix S1, Eqn 17). However, many sound level meters apply standardised filters known as A- and C-weightings, which modify the signal to approximate the frequency response of human hearing (Kinsler *et al*. 1999). It is therefore important to consider whether the frequency range of interest coincides with human audibility (which it may, e.g. Brumm 2004) and whether a human frequency-weighted metric is appropriate. Some sound level meters have an unweighted setting (known as Z-weighting, flat or linear), but are still limited to the nominal frequency range of human hearing.

**Table 2:** Units and abbreviations for quantities described in the text. Note that some authors have used units of dB re *p*_*ref*_ *μ*Pa^2^ for SPL and TOLs: this notational difference does not affect the numerical levels reported

Term	Abbreviation	Units in air	Units underwater	Short definition
Sound pressure level	SPL	dB re 20 *μ*Pa	dB re 1 *μ*Pa	Sound level over specified frequency range as a single number
Power spectral density	PSD	dB re (20 *μ*Pa)^2^Hz^−1^	dB re 1 *μ*Pa^2^Hz^−1^	Standardised spectrum of sound levels across frequency
1/3-octave band level	TOL	dB re 20 *μ*Pa	dB re 1 *μ*Pa	Coarse sound level spectrum with logarithmic frequency scaling
Sound exposure level	SEL	dB re (20 *μ*Pa)^2^s	dB re 1 *μ*Pa^2^s	Cumulative measure of sound energy

These limitations of the sound level meter can be avoided by instead making calibrated sound level measurements using portable or autonomous field recorders. This has long been standard practice in aquatic bioacoustics (Au & Hastings 2008), where portable field recorders are used to record data from a hydrophone lowered over the side of a drifting vessel. Unlike data from sound level meters, field recordings also enable post hoc analyses to identify components of the acoustic environment or to produce other acoustical metrics. Calibrating the recordings requires knowledge of the relevant hardware specifications and an understanding of the signal processing steps required for the calibration procedure (Appendix S1, Section 3). Although such calibrated measurements of terrestrial acoustic habitats have been made (Waser & Brown 1986), these are the exception: habitat monitoring studies more commonly report relative (i.e. uncalibrated) sound spectra (Boinski & Campbell 1995), sometimes supplemented by measurements made with sound level meters (Lengagne & Slater 2002).

Portable field recorders are well suited to short-term surveys in favourable weather conditions. A battery-powered digital audio recorder can be rapidly deployed with a microphone mounted on a hand-held boom or tripod, lashed to vegetation, or with a hydrophone lowered to a desired depth in the water. Care should be taken to minimise incidental noise from the presence of the monitoring platform and noise generated by flow of air or water past the acoustic sensor (known as flow noise or ‘pseudonoise’).

**Fig 1:**
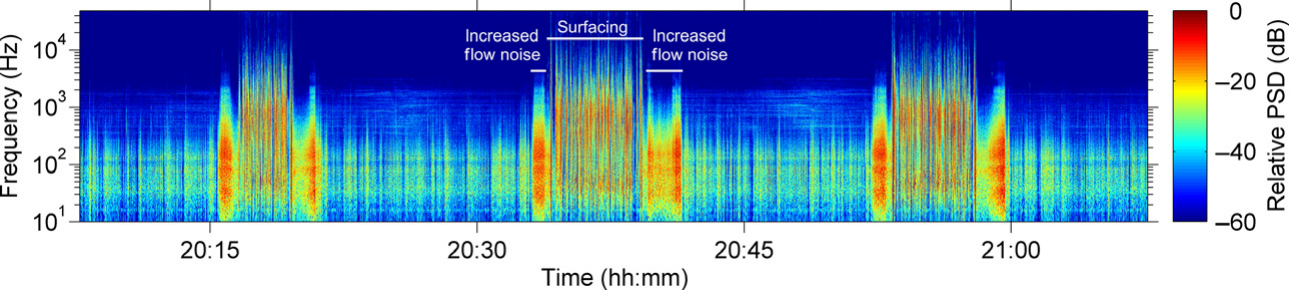
Recording from an acoustic recording tag (DTAG) attached to a North Atlantic right whale in the Bay of Fundy, Maine, USA, on 3 August 2005. Sampling rate: 96 kHz. Note the periodic surfacing events (three in total) and the increase in flow noise before and after these surfacing events caused by increased travel speed.

Other mobile platforms have largely been developed for marine bioacoustics applications. For example, to study marine mammal behaviour, acoustic tags have been developed which are temporarily attached to animals via suction cups, recording sound and tracking movement for up to several days (Burgess *et al*. 1998; Johnson & Tyack 2003). While these devices have been effective in studying vocalisations in cetaceans (Johnson *et al*. 2004) and exposure to high-amplitude anthropogenic noise (DeRuiter *et al*. 2013), the ability to record lower-amplitude low-frequency sound is limited: the movement of the animal through the water causes turbulence around the hydrophone, producing flow noise which contaminates low frequencies (Johnson & Tyack 2003), and acoustic time series are periodically disrupted by surfacing events (Fig.1) and vocalisations of the host animal. Application of similar recording tags to terrestrial wildlife has thus far been limited to one study of wild mule deer (*Odocoileus hemionus*; Lynch *et al*. 2013). Emerging mobile platforms also include autonomous underwater gliders (Rudnick, Davis & Eriksen 2004; Baumgartner & Fratantoni 2008), which can be deployed for up to several hundred days, and freely drifting platforms (Wilson, Benjamins & Elliott 2013), which minimise flow noise in high tidal flow environments by moving with the current.

### General considerations

The ability of PAM systems to accurately record sound is limited by several factors: dynamic range, frequency range and system self-noise. The dynamic range is the ratio of the highest to the lowest amplitude that can be measured by a given system and can be scaled to higher or lower amplitudes by adding gain to the signal (within the limitations of the device's own self-noise; Johnson, Partan & Hurst 2013; Merchant *et al*. 2013). If the gain is too low, quieter sounds may not be recorded (Rempel *et al*. 2013), and if it is too high, loud sounds can saturate the system, leading to distortion of the signal through clipping (Madsen & Wahlberg 2007; Fristrup & Mennitt 2012). A PAM system should be chosen whose frequency range encompasses the spectral content of all sounds of interest to the study. This range is limited to half the sampling rate of the recordings (the Nyquist frequency), and by the sensitivity of the transducer and any filtering in the recording system (Appendix S1; Madsen & Wahlberg 2007). System self-noise is noise generated by the recording system and acoustic transducer and can limit the ability of a system to record low-amplitude sound (Fristrup & Mennitt 2012; Johnson, Partan & Hurst 2013). It is therefore important to consider self-noise specifications when selecting a PAM system, and where possible to measure self-noise levels by making recordings in a quiet location isolated from sources of vibration.

A wider consideration is that many species (e.g. many fish, insect and arthropod species) primarily sense sound not through sound pressure, but through particle motion, a directional property of the sound field (Hawkins 1981; Stumpner & von Helversen 2001). The devices described above do not directly measure particle motion, which may be significant in the region near to sound sources (the near field) or close to reflecting surfaces, where variations in sound pressure and particle motion can differ considerably (far from the source and surfaces – in the far field – sound pressure and particle motion are directly related). Although many studies have measured auditory sensitivity to particle motion in controlled experiments, the use of particle motion sensors in habitat monitoring is still in its infancy.

## Data analysis

There is no standard or widely available software for the production of calibrated PAM measurements. While there are several options for annotation, detection and classification of bioacoustic signals [e.g. Raven (Charif, Clark & Fristrup 2004), PAMGuard (Gillespie *et al*. 2009)], these operations do not demand absolute measures of sound level and so typically use relative levels of signal amplitude. Calibrated measurements require hardware-specific data (the microphone or hydrophone sensitivity and properties of the data acquisition system), and many research groups use custom-developed programs for PAM analysis tailored to their equipment. In the accompanying tutorial (Appendix S1), we present a detailed guide to the signal processing steps required to produce calibrated PAM data in terrestrial and aquatic environments, including *PAMGuide* (Data S1), a template code provided in matlab and r versions which implements the equations presented in the tutorial. Here, we present a non-technical outline of the calibration procedure and a description of key acoustical metrics. Application of these metrics to the characterisation of acoustic habitats is illustrated in the following section.

**Fig 2:**
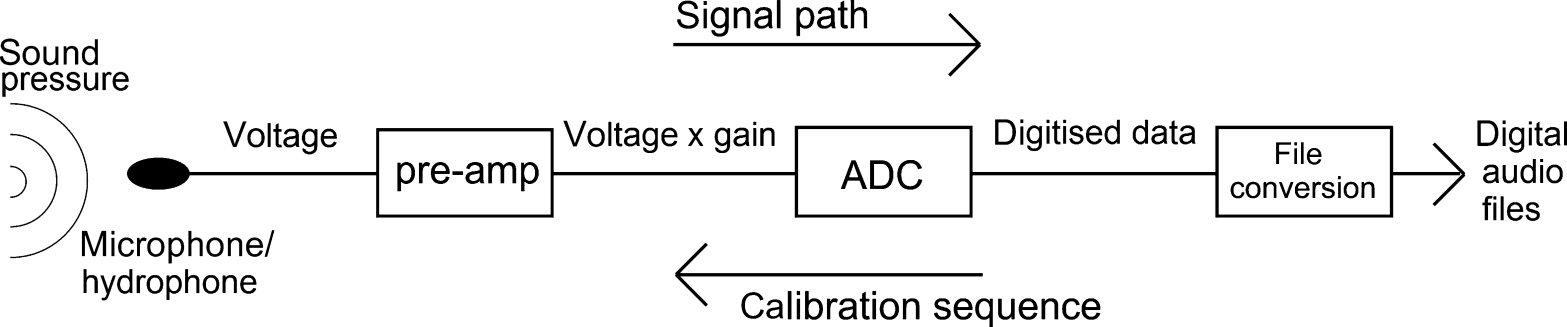
Signal path and calibration sequence for a typical passive acoustic monitoring system. ADC, analogue-to-digital converter.

The basic principle of making absolute sound pressure measurements is to reverse the transformations made to the signal along its path into the data acquisition system (Fig.2). The same steps apply to in-air and underwater PAM devices, which employ the same fundamental components: (i) an acoustic transducer (microphone or hydrophone) to convert the sound pressure into a voltage signal; (ii) a pre-amplifier, which is used to increase the amplitude of the voltage signal before it is recorded and may apply frequency equalisation and anti-aliasing filters; and (iii) an analogue-to-digital converter (ADC), which digitises the analogue voltage signal and then packages the data as audio files with a standardised amplitude range (Au & Hastings 2008; e.g. a 16-bit recording has an amplitude range of ±2^16−1^ bits, i.e. integers between −32 768 and 32 767). To retrieve the original measurement of sound pressure from the audio files, it is therefore necessary to know: (i) the sensitivity of the acoustic transducer, which defines how much voltage is generated per unit of sound pressure; (ii) the amount of voltage gain (if any) applied by the pre-amplifier; and (iii) the ADC input voltage which corresponds to the maximum amplitude that can be represented in the audio files (Robinson, Lepper & Hazelwood 2014). This process is summarised in Fig.2. Alternatively, an ‘end-to-end’ calibration can be carried out by inputting a known signal into the transducer, for example with a pistonphone (Appendix S1, Section 3). It is important to note that the transducer sensitivity and the gain of the pre-amplifier may vary significantly with frequency, in which case frequency-dependent corrections should be applied to the signal.

This procedure yields the relationship between the recorded signal and the signal at the transducer, allowing the sound pressure signal (known as the pressure waveform) to be computed from the recordings. The pressure waveform is used to measure the amplitudes of impulsive sounds, such as echolocation calls or gunshots (see next section). Acoustic habitat characterisation generally requires further analysis of the pressure waveform, such as transformation into the frequency domain to analyse frequency characteristics and averaging to represent longer periods (Appendix S1). The output of these analyses is typically one of a number of common acoustical metrics shown in Table 2.

**Fig 3:**
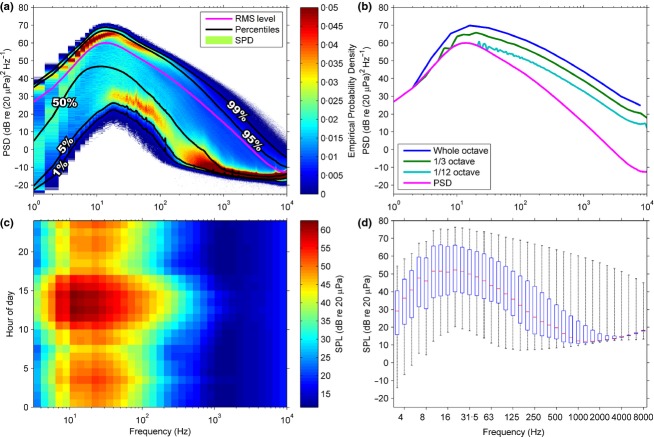
Statistical analyses of long-term acoustic data recorded using a Wildlife Acoustics SM2+ SongMeter at the Central Plains Experimental Research Station, Colorado, from 19 October to 13 December 2013. Sampling rate: 44.1 kHz. (a) RMS level of the PSD, percentiles and SPD, showing bimodality of sound level distribution and presence of noise floor above ∼100 Hz (b) RMS level of the PSD and fractional octave bands (c) Median 1/3-octave level for each hour of the day. (d) Boxplot of 1/3-octave bands – mid-line is median, edges of boxes are first and third quartiles, whiskers are minima and maxima. Note that the noise floor of the instrument limits the range of the higher frequency bands.

Broadband sound pressure level (SPL) is the most ubiquitous acoustic metric and expresses the root-mean-square (RMS) sound amplitude within a given time window and frequency range as a single decibel (dB) level, for example 64 dB re 20 *μ*Pa (Kinsler *et al*. 1999). Most acoustical metrics are expressed as a decibel level relative to a reference pressure, *p*_*ref*_, in the form X dB re *p*_*ref*_. In air, *p*_*ref*_ is 20 *μ*Pa, which corresponds to the nominal threshold of human hearing at 1 kHz, while in underwater, *p*_*ref*_ is 1 *μ*Pa. Spectra showing how sound level varies with frequency are given by the power spectral density (PSD), which describes the power in the equivalent of 1-Hz bands in the frequency domain (although the actual frequency resolution may be much coarser than 1 Hz), or by fractional octave analysis (typically 1/3-octave band levels, TOLs), which measures the power in frequency bands that widen exponentially with increasing frequency and are evenly spaced on a logarithmic frequency axis (Fig.3c). Finally, the sound exposure level (SEL) is a summation of sound energy through time over a specified duration and is often used to assess cumulative exposure to noise.

An emerging area of study is the use of acoustic indices as proxy indicators of biodiversity and species composition (Towsey, Parsons & Sueur 2014). Various new statistical indices (e.g. acoustic diversity index, acoustic complexity index) have been applied in comparative studies of distinct habitats for this purpose, so far with mixed results (Lellouch *et al*. 2014; Towsey, Parsons & Sueur 2014). It remains to be seen to what extent such techniques will complement field observations of biodiversity and automatic detection and classification of species via PAM.

## Habitat characterisation

Acoustic habitats can be described by statistical analysis of sound levels and by time-series representations. Based on the metrics outlined above and defined in Appendix S1, this section illustrates how these techniques can be used effectively for various habitat monitoring applications. In general, statistical analyses are more suited to characterising variability and comparing acoustic habitats at differing times or locations, while discrete events and trends in sound levels are better described by time series.

### Statistical analysis

There are several ways to calculate the average sound level from a series of shorter measurements, each of which has particular advantages. The most common metric is the RMS level (see Fig.3), which is the mean of the squared sound pressure computed before it is converted to dB (Appendix S1, Eqn 18). The RMS level is the most prevalent averaging metric, perhaps due to the historic centrality of *L*_eq_ in terrestrial noise characterisation (*L*_eq_ is the equivalent RMS level during a specified period, which correlates with human noise disturbance), and its direct relation to SPL, which makes it independent of the length of the time segments used in the original analysis (unlike other averaging metrics; Merchant *et al*. 2012). However, the RMS level is also strongly influenced by the highest sound levels (it can be >95th percentile in some cases; Merchant *et al*. 2013) and so should be used with caution if applied to recordings with intermittent high-amplitude events (e.g. pile driving; Madsen 2005).

Alternative averages can give more statistically representative measures of sound levels than the RMS level. The mode, defined as the sound level corresponding to the maximum probability density at each frequency, is (by definition) the most representative metric, although few studies have made use of it (e.g. Parks, Urazghildiiev & Clark 2009; Merchant *et al*. 2012) and there is the potential for multimodality to produce misleading results (Fig.3a; Merchant, *et al*. 2012, 2013). Median sound levels (Fig.3d; known as *L*_50_ in terrestrial noise assessment) can also be used as an indicator of typical sound levels in a habitat (e.g. Klinck *et al*. 2012). These are more robust than the mode and are generally insensitive to limitations in the dynamic range of the recording instrument.

The range of sound levels in a habitat can be assessed by plotting the percentile levels across the frequency spectrum (Fig.3a; Richardson *et al*. 1995; Castellote, Clark & Lammers 2012). These are alternatively known as exceedance levels, although the percentiles are reversed, for example the 95% exceedance level, *L*_95_, is equivalent to the 5th percentile. Percentiles provide an approximate indication of the distribution of sound levels and may be useful in characterising the potential extent of acoustic masking for a particular species (Clark *et al*. 2009). A more comprehensive analysis of the sound level distribution is given by the spectral probability density (SPD; Merchant *et al*. 2013), whereby the empirical probability density of sound levels in each frequency band is presented (Fig.3a). This shows the modal structure and outlying data in the underlying distribution, which helps to interpret averages and percentiles. It can also reveal limitations in the recording system: for example, in the SPD shown in Fig.3a, the self-noise of the instrument appears to limit recording of the lowest sound levels in the habitat above *c*. 100 Hz, evidenced by the flattening gradient of the lowest data points and their convergence with the mode above this frequency.

Other statistical analyses include average levels for particular temporal periods to examine cyclical trends, such as diel, seasonal or annual variability (see Fig.3c, although other configurations are also used, e.g. Radford *et al*. 2008), and box-and-whisker plots (Fig.3d; Bassett *et al*. 2012) showing the spread of quartiles in each band.

In the frequency domain, fine-scale variations in the sound spectrum can be assessed using the PSD, which is often computed at 1-Hz resolution (Fig.3b), although this can be computationally demanding when processing and storing large data sets. Frequency resolution can be reduced using either coarser PSDs or fractional octave analysis, most commonly in 1/3-octave bands (Appendix S1, Eqns 13–16), wherein the frequency range of the band is directly proportional to the centre frequency (constant Q). For some taxa (e.g. mammals), constant Q frequency bands are a particularly useful tool for acoustic habitat analysis, as they can approximate the response of the auditory system. It is important to note that the spectral slope of the fractional octave band levels differs from the PSD (Fig.3b), as the bands are scaled logarithmically with frequency (i.e. they widen with increasing frequency), meaning that higher frequency bands integrate energy over larger frequency ranges. For this reason, spectra with differing frequency bandwidths (e.g. PSD and 1/3-octave) cannot be directly compared.

### Time series

Time series are used to characterise discrete events, such as vocal behaviour or anthropogenic noise events (typically in the form of spectrograms), and to track temporal trends in sound levels, usually in particular frequency bands (e.g. TOLs or broadband level).

**Fig 4:**
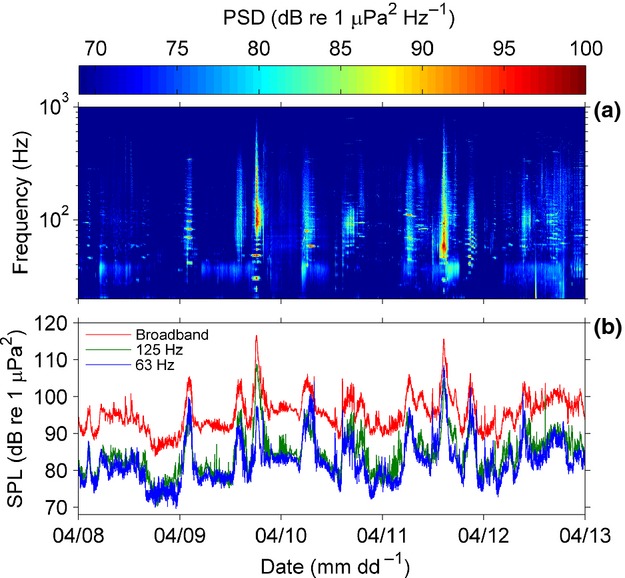
Time-series analysis of ship passages recorded using an autonomous marine acoustic recording unit (MARU; developed by the Bioacoustics Research Program at Cornell University) in Stellwagen Bank National Marine Sanctuary, Massachusetts Bay, USA, in April 2010. Sampling rate: 2 kHz. (a) Spectrogram composed of PSDs with 1-s time segments; (b) 63- and 125-Hz 1/3-octave bands and broadband (0.2–1 kHz) SPL. Analysis parameters: 1-s time segments averaged to 60-s resolution via the Welch method.

A widely used time-series plot is the spectrogram, which comprises a series of PSD measurements showing how the sound level varies with time at each frequency (Fig.4a). Sound sources can then be identified by visual inspection and by listening to the sound files (depending on the frequency range), or by automatic detection and classification. The time and frequency resolution of the spectrograms must be sufficiently high to resolve the sound events under consideration, and overlapping time windows may be used to smooth data in the time domain and to ensure sounds at the boundary between time windows are represented (Appendix S1, Section 4).

As well as describing discrete events, time series are used to track trends in sound levels in particular frequency bands (Fig.4b), often in the context of long-term anthropogenic noise studies (e.g. Miksis-Olds, Bradley & Niu 2013).

**Fig 5:**
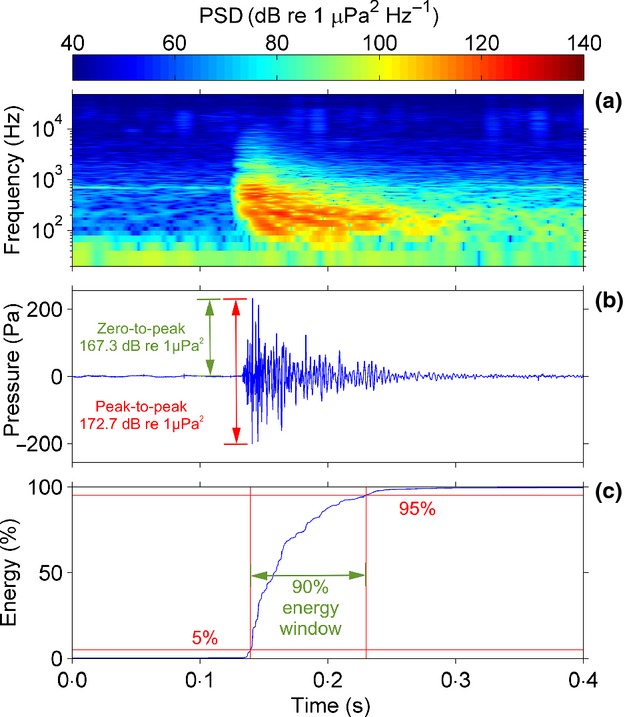
Seismic airgun array recorded at 6 km distance using a Wildlife Acoustics SM2M deployed off southern Gabon, West Africa, 30 October 2012. Sampling rate: 96 kHz. (a) PSD: 0.05-s Hann window, 99% overlap. (b) Pressure waveform illustrating peak-to-peak and zero-to-peak metrics. (c) Cumulative energy of pulse showing 90% energy envelope.

While the amplitudes of nominally continuous sounds (e.g. tonal vocalisations, ships, wind-generated sound) can be accurately represented in frequency spectra, this is not the case for impulsive sounds (e.g. echolocation calls, snapping shrimp, gunshots, seismic airguns, pile driving; Fig.5a). If impulsive sounds are identified, their amplitudes should instead be measured using the pressure waveform (Fig.5b), which gives a much finer time resolution with which to measure the impulse amplitude (Madsen 2005). The peak amplitude of the pulse is described by either the peak-to-peak (SPL_*p*−*p*_) or zero-to-peak pressure (SPL_0−*p*_), as illustrated in Fig.5b (Appendix S1, Eqns 22–23). A further metric, sound exposure level (SEL) describes the acoustic energy contained in the pulse (Appendix S1, Eqns 24–25). The duration of the pulse is commonly defined by the 90% energy envelope: the time window between 5% and 95% of the cumulative acoustic energy (Blackwell, Lawson & Williams 2004; Fig.5c). To accurately measure acoustic pulses, a recording system should be chosen whose sensitivity does not vary significantly within the peak frequency range of the pulse, and the signal-to-noise ratio (SNR) of the pulse should be ≥ 10 dB (Appendix S1; Madsen 2005; Madsen & Wahlberg 2007).

## Future directions

The temporal range of acoustic monitoring has been greatly increased by advances in instrumentation and data analysis. Looking forward, the emerging area of acoustic habitat mapping promises in turn to expand the spatial scope of monitoring studies. Integrating PAM data with spatial models has the potential to produce ground-truthed sound maps of acoustic habitats (Barber *et al*. 2011; Erbe, MacGillivray & Williams 2012; NOAA 2012; Mennitt, Sherrill & Fristrup 2014), which with large-scale monitoring could extend to the regional and national scales relevant to ecosystem-scale assessment (Barber *et al*. 2011; Mennitt, Sherrill & Fristrup 2014) and the migratory ranges of wide-ranging species (NOAA 2012). If such maps are sufficiently predictive of sound levels, they could help to highlight areas of concern for anthropogenic noise impact when overlain with animal distributions, and offer new perspectives on the spatial ecology of acoustically sensitive species. Large-scale mapping inevitably involves pooling measurements made by many groups, potentially using different devices; thus, the success of these modelling efforts will depend in part on the quality and standardisation of passive acoustic measurements they are based on. We hope that the overview of methods presented here contributes to a robust methodological foundation for this progress and helps to make passive acoustic techniques more accessible to newcomers to the field.

## Data accessibility

matlab and r codes used in the example analyses are available in the Supporting Information.

## Supporting Information

**Appendix S1.** PAMGuide tutorial.

**Data S1.** PAMGuide.zip - Zipped archive of R and MATLAB codes for PAMGuide.
