# Supplementary material for: Measuring acoustic habitats
Source: Methods Ecol Evol. 2015 Jan 27;6(3):257–65. doi: 10.1111/2041-210X.12330 (PMC4413749; doi:10.1111/2041-210X.12330)
Supplement: Supplementary file 1 — Appendix S1. PAMGuide tutorial. [file mee30006-0257-sd1.pdf]

Measuring acoustic habitats
Appendix S1

Nathan D. Merchant, Kurt M. Fristrup, Mark P. Johnson, Peter L. Tyack, Matthew J. Witt, Philippe Blondel, Susan E. Parks

Methods in Ecology and Evolution

This appendix is a tutorial on how to analyse passive acoustic data to produce measurements of acoustic habitats using *PAMGuide*, the software that accompanies the above article and which can be run in MATLAB or R. Statistical analysis of passive acoustic data and the application of metrics to different monitoring scenarios are discussed in the *Habitat characterisation* section of the main text.

Contents

1 Introduction **3**

2 Quick start guide **4**

2.1 Initialising *PAMGuide* 4
2.2 Running *PAMGuide* 5
2.3 Calibrated analysis 7
2.4 Verifying *PAMGuide* output using test files 9
2.5 Adding time stamps 11
2.6 Analysing large files 13
2.7 Batch processing 13
2.8 *Viewer* 15

3 Calibrating data **16**

3.1 Implementation 16

3.2 Background 18

4 Selecting analysis parameters **21**
4.1 Analysis type 21
4.2 Window type 22
4.3 Window length 23
4.4 Window overlap 24
4.5 Low- and high-frequency limits 25
4.6 Averaging data via the Welch method 26

5 Plot types **27**

6 Technical background **29**
6.1 Computing frequency spectra 29
6.2 TOLs and broadband analysis 32
6.3 Statistical metrics 33
6.4 Reducing time resolution 34
6.5 Computing the acoustic waveform 35
6.6 Impulse metrics 35
6.7 Sound exposure level 35
6.8 Signal-to-noise ratio and the system noise floor 36
6.9 Tonal signals 38

7 *PAMGuide* index **39**
7.1 MATLAB 39
7.2 R 41

References **42**

1 Introduction

The tutorial is structured in three parts:

1. A quick start guide (Section 2), which gives straightforward instructions on getting started with *PAMGuide*.

2. Detailed instructions on calibrating data (Section 3), selecting analysis parameters (Section 4), and available plot types in *PAMGuide* (Section 5).

3. Technical background (Section 6), which provides the underlying signal processing steps and equations that *PAMGuide* uses.

For reference, Fig. 1 summarises the workflow of *PAMGuide* and the plots produced for each output metric. An index of the parameters in *PAMGuide* is provided in Section 7.

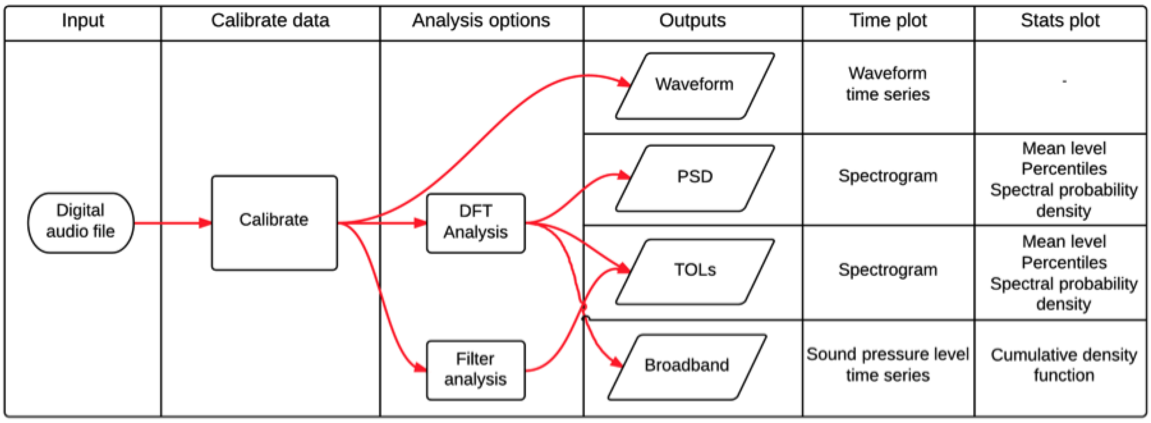

Figure 1: Flow diagram for *PAMGuide* illustrating processing steps and plot types corresponding to each output metric. DFT = discrete Fourier transform; PSD = power spectral density; TOL = 1/3-octave band level.

2 Quick start guide

The section covers the basics to get started with analysing sound files in *PAMGuide*. Subsequent sections contain detailed discussion of calibration procedures (Section 3, p16), selection of analysis parameters (Section 4, p21), and plotting options (Section 5, p27).

Table 1: Inventory of *PAMGuide* files.

**MATLAB**		**R**
PAMGuide.m	PG_TOL.m	PAMGuide.R
PAMGuide.fig	PG_Viewer.m	Meta.R
PG_Func.m	PG_Waveform.m	PAMGuide_Meta.R
PG_DFT.m		Viewer.R
**Test files**		
WhiteNoise_10s_48kHz_+-0.5.wav	Sine_10s_48kHz_+-0.5.wav	

2.1 Initialising *PAMGuide*

Launch MATLAB or R.

**MATLAB**	**R**
Set current folder to folder containing *PAMGuide* scripts:<br><br>**`>> cd('`***folderpath***`')`**<br><br>Launch *PAMGuide*:<br><br>**`>> PAMGuide`**<br><br>*PAMGuide* graphical user interface (GUI) will appear (see Fig. 6, p40).	Set current folder to folder containing *PAMGuide* scripts:<br><br>**`> setwd('`***folderpath***`')`**<br><br>Initialise *PAMGuide*:<br><br>**`> source('PAMGuide.R')`**<br><br>This command loads the *PAMGuide* function into the workspace, meaning it can then be called from the command line. Note: if not already installed, R will install package 'tuneR' on first execution.

The current folder ***must* be set to the *PAMGuide* folder** for *PAMGuide* to operate.

2.2 Running *PAMGuide*

**MATLAB**	**R**
Click **Select file...**. A dialogue box will appear. Select the WAV file for analysis. AIFF files can also be analysed if using MATLAB R2014 or later.<br>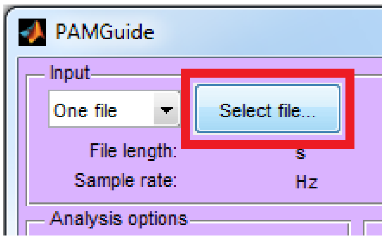 	Call *PAMGuide*:<br>`> PAMGuide()`<br>A dialogue box will appear. Select WAV file for analysis. *PAMGuide* will then display settings and analysis progress in the command line.
Click **RUN**. By default, *PAMGuide* will carry out a PSD analysis, and then plot the time representation (spectrogram) and statistical representations of the data. This uncalibrated analysis is plotted in relative units. For absolute levels, calibration data must be input (see Section 3, p16).<br>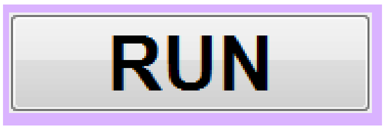 	By default, *PAMGuide* will carry out a PSD analysis, and then plot the time representation (spectrogram) and statistical representations of the data. This uncalibrated analysis is plotted in relative units. For absolute levels, calibration data must be input (see Section 3, p16).

Continued over...

<table>
<tr>
<td>

Other analysis types (e.g. broadband level, waveform) are selected in the **Analysis type** drop-down menu, and analysis parameters defined using the other inputs in the **Analysis options** panel. These options are discussed in detail in Section 4, p21.

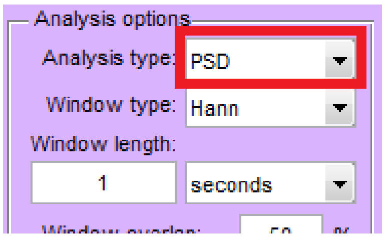

</td>
<td>

Other analysis options can be selected by specifying parameters when calling *PAMGuide*. For example:

> PAMGuide(atype=”13oct”, envi="Wat”,N=2048)

specifies a 1/3-octave band analysis, an underwater measurement, and a window length of 2048 samples, and the default parameters for all others. A full list of these parameters and their defaults is provided in Section 7, p39, and guidance on selecting parameters for different purposes is provided in Section 4, p21.

</td>
</tr>
<tr>
<td>

Plotting of time or statistical representations can be selected using the **Plot type** drop-down menu. Options: **Both**/**Time**/**Stats**/**None**. Default is **Both**.

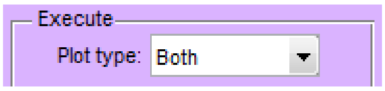

See Section 5, p27, for examples of each plot type.

</td>
<td>

Plotting of time or statistical representations can be selected using **plottype**. Options: **Both**/**Time**/**Stats**/**None**. Default is **Both**. E.g.:

> PAMGuide(plottype=”Stats”)

See Section 5, p27, for examples of each plot type.

</td>
</tr>
<tr>
<td>

Figures can be saved from the figure window. Click **File>Export setup...**, then in the **Rendering** tab, choose resolution (300 dpi or greater for publishing quality), and click **Export...**. Image format can be chosen in the subsequent dialogue box.

</td>
<td>

Figures can be saved as PDF files using **Files>Save As...**

</td>
</tr>
</table>

Continued over...

PAMGuide can output analysis files in CSV format for subsequent use by selecting **Write output data to file** in the **Execute** panel.<br>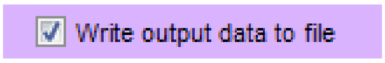<br><br>These files can be read and time and statistical representations plotted using *Viewer* (see Section 2.8). The format of the output files is a heading row with the frequencies of each of the columns, while the first column contains the times of each of the rows, with the data commencing on the second row of the second column. The first cell in the first column stores metadata on the analysis parameters, which is used by *Viewer*.	*PAMGuide* can output analysis files in CSV format for subsequent use using **outwrite**. Default is 0 (i.e. no output file written). Output file is written to same directory as input file.<br>`> PAMGuide(outwrite=1)`<br>These files can be read and time and statistical representations plotted using *Viewer* (see Section 2.8). The format of the output files is a heading row with the frequencies of each of the columns, while the first column contains the times of each of the rows, with the data commencing on the second row of the second column. The first cell in the first column stores metadata on the analysis parameters, which is used by *Viewer*.

2.3 Calibrated analysis

For calibrated analyses, units will be expressed as absolute units, and relative to the relevant reference pressure if expressed in decibels (see Table 1 of main text).

MATLAB	R
Select **Calibrate data** in the **Calibration** panel:<br>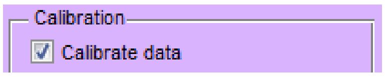 	Set calibration parameter, **calib**, to 1:<br>`> PAMGuide(calib=1)`

Continued over...

Select **Domain** (**In Air**/**Underwater**): 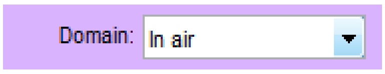	Specify the environment parameter, **envi**, if an underwater measurement (default is **Air**)<br><br>`> PAMGuide(calib=1,envi="Wat")`
Choose **Calibration type**. Options: **Tech. specs**/**End-to-end**/**Recorder + Microphone/hydrophone**: 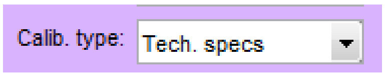 These options are discussed in detail in Section 3 (p16).	Specify calibration type, **ctype** (see Section 3 for details). Default is **MF** (manufacturer's technical specifications):<br><br>`> PAMGuide(calib=1,envi="Wat", ctype="MF")`
Define calibration parameters. For example, if using the manufacturer's specifications plus the gain settings used in the deployment, select **Tech. specs** in the **Calib. Type** drop-down menu and input the specifications below. For a Wildlife Acoustics SM2+ in air with gain set to +24 dB, the calibration panel options would be: 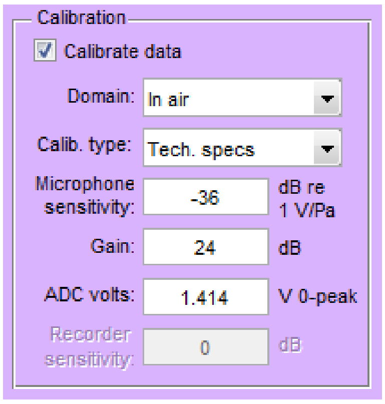	Define calibration parameters (see Section 3 for details). E.g. for in-air SM2+ recording with user-defined gain set to +24 dB:<br><br>`> PAMGuide(calib=1,envi="Air", stype="MF",mh=-36,g=24, vADC=1.414)`

Continued over...

<table>
<tr>
<td>For an underwater measurement using a Wildlife Acoustics SM2M and user-defined gain of +12 dB, the parameters would be:
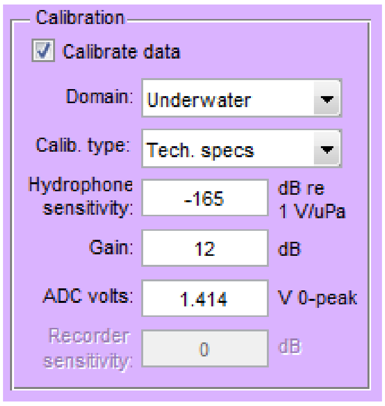
</td>
<td>For an underwater measurement using a Wildlife Acoustics SM2M and user-defined gain of +12 dB, the parameters would be:

> PAMGuide(calib=1,envi=”Wat”,
stype=”MF”,mh=-165,g=12,
vADC=1.414)</td>
</tr>
</table>

2.4 Verifying *PAMGuide* output using test files

Two test files are supplied with *PAMGuide* which users can use to verify that the absolute levels produced by *PAMGuide* are correct: **WhiteNoise_10s_48kHz_+-0.5.wav** and **Sine_10s_48kHz_+-0.5.wav**. The verification process for each file is described below.

White noise

The first test file is a 10-second sample of white noise (random noise with a flat frequency spectrum): **WhiteNoise_10s_48kHz_+-0.5.wav**.

Using the following settings, the RMS level of the white noise test file should be 197.0 dB re 1 μPa. This metric is output in the command line during analysis as: `RMS level (mean SPL) = 197.0 dB re 1 uPa.`

In MATLAB, replicate the settings in Fig. 2; in R, use:

```
> PAMGuide(atype="Broadband",calib=1,envi="Wat",Mh=-200,
vADC=2,plottype="Time")
```

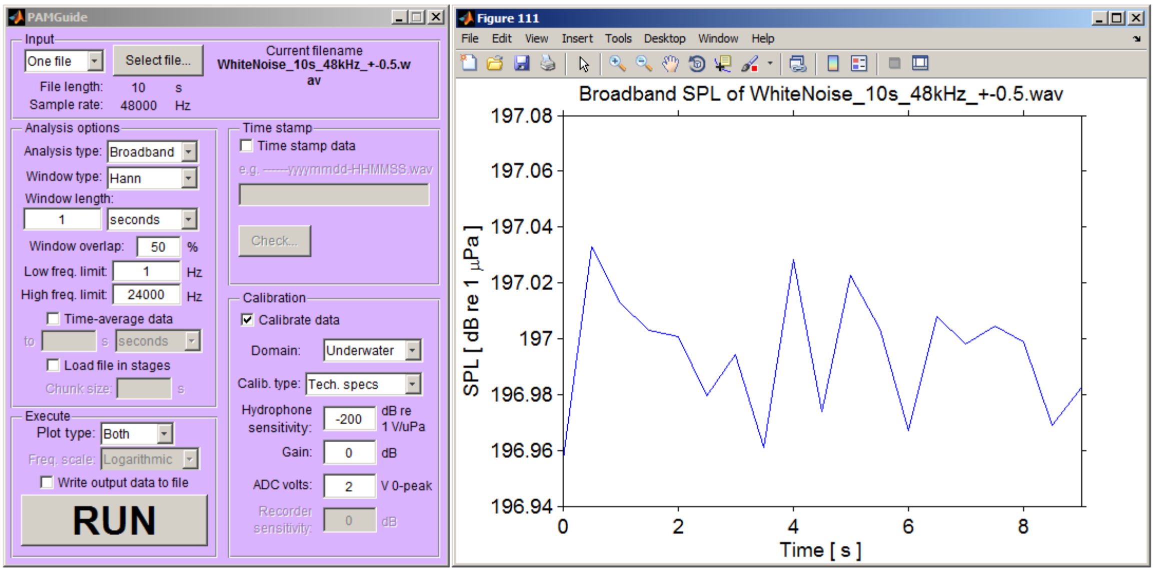

Figure 2: Settings and output for white noise verification file.

Sine wave

The second test file is a 10-second sine wave, or pure tone, at a frequency of 1 kHz: **Sine_10s_48kHz_+-0.5.wav**. For reasons explained in Section 6.9 (p38), the correct analysis to determine the amplitude of a pure tone is the power spectrum, which is closely related to the PSD (see Section 6.1, p29).

Using the following settings, the power spectrum amplitude of the sine wave test file should be 197 dB re 1 μPa^2 Hz^{-1} at 1 kHz. In MATLAB, replicate the settings in Fig. 3; in R, use:

```
> PAMGuide(atype="PowerSpec",calib=1,envi="Wat",Mh=-200,
vADC=2,plottype="Stats")
```

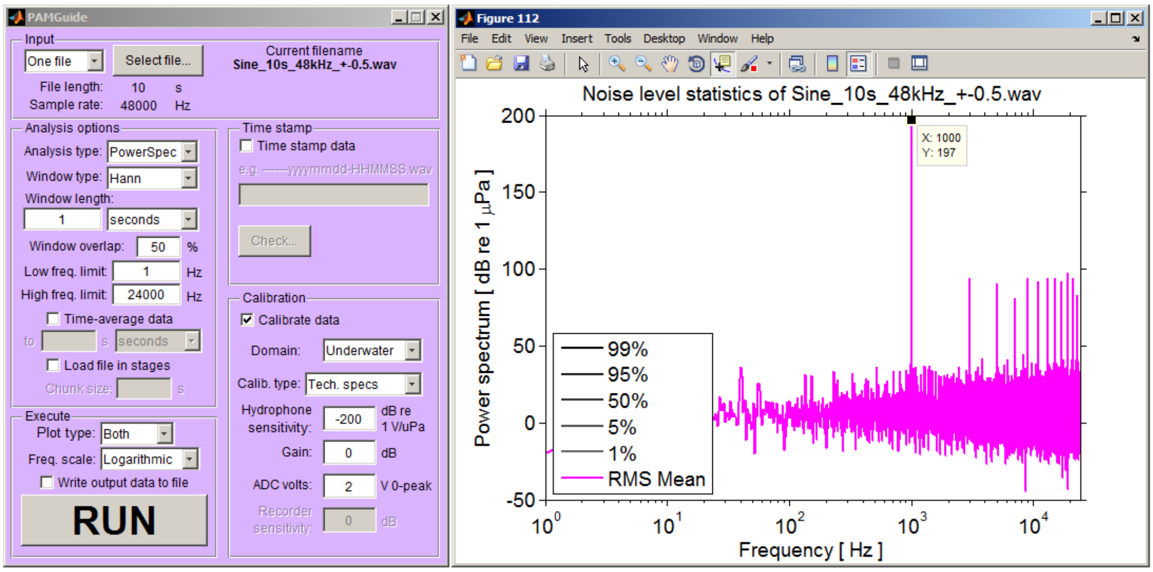

Figure 3: Settings and output for 1 kHz sine wave verification file.

2.5 Adding time stamps

Most autonomous passive acoustic recorders will embed a time stamp in the file name of each audio file. This information can be extracted in *PAMGuide* to produce analyses with absolute time axes. If time stamps are added, time series will be plotted with each file beginning at the time declared in the time stamp.

**MATLAB**	**R**
Select **Time stamp data** in the **Time stamp** panel: 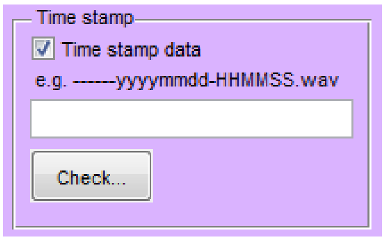	Time stamping is implemented if **timestring** is defined when *PAMGuide* is called. E.g.<br><br>**`MyRecording_Dep1456_20140707_141503.wav`**<br><br>becomes:

Continued over...

In the text entry box, enter the name of the analysis file with digits corresponding to the time stamp replaced with letters (**y** = year, **m** = month, **d** = day, **H** = hour, **M** = minute, **S** = second, **F** = millisecond), and all other characters replaced with dashes, ‘**-**’. E.g.

```
MyRecording_Depl456_20140707_141503.wav
```

becomes

```
--------------------yyyymmdd-HHMMSS.wav
```

```
> PAMGuide(timestring="MyRecording_Depl456_%Y%m%d_%H%M%S.wav")
```

where %**Y** = year, %**m** = month, %**d** = day, %**H** = hour, %**M** = minute, %**S** = second.

When batch processing multiple files (see Section 2.7), file names must have the same prefix before the time stamp.

If you have already selected a file, its name should be displayed in the **Input** panel. To check that the time stamp format is correct, click **Check...**, and the date and time will be displayed to the right, or else **ERROR: Check format** if the format cannot be interpreted.

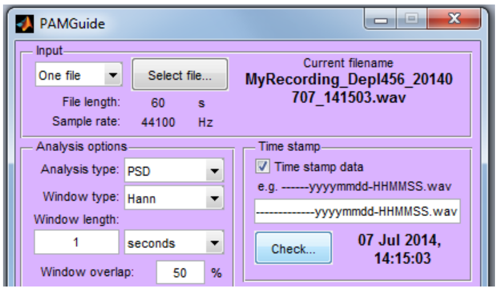

2.6 Analysing large files

A common problem with analysing large sound files is that the host computer may not have enough RAM to load and analyse an entire file at once. To get around this, *PAMGuide* has the option to load and analyse files in stages. *PAMGuide* then outputs the plots and output files of the entire file as usual.

MATLAB	R
Select **Load file in stages** in the **Analysis options** panel:<br>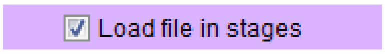<br><br>Specify a chunk size in seconds by which to subdivide the input file:<br>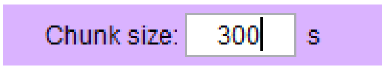 	Specify the chunk size, **chunksize**, in seconds when calling *PAMGuide*:<br>`> PAMGuide(chunksize=300)`

2.7 Batch processing

PAMGuide can batch process multiple files, and will produce contiguous plots of multiple files from the same deployment provided the sampling rate of the files (e.g. 44100 Hz) is the same for all files. Place all audio files for analysis in one folder.

MATLAB	R

Continued over...

<table>
<tr>
<td>
Select Batch from the drop-down menu in the Input panel:

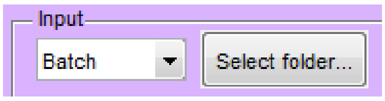

Click Select folder... and select the folder for analysis in the dialogue box.

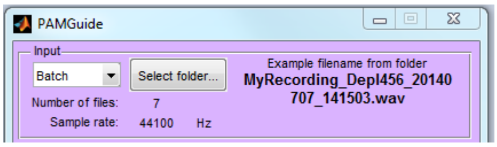

The name of the first file in the selected folder will appear in the Input panel under Example filename from folder. This is the file that will be used for testing the time stamp format (see Section 2.5), if selected. The other settings used in the analysis can be defined as before. Then click RUN.
</td>
<td>
In R, batch processing is achieved using Meta.R, contained in the PAMGuide folder.

Initialise Meta:

<code>> source('Meta.R')</code>

The analysis settings for Meta are input in the same way as PAMGuide.

Run Meta:

<code>> Meta()</code>

A dialogue box will appear. Select any file in the folder you wish to analyse, and Meta will gather file names for the whole folder.
</td>
</tr>
<tr>
<td>
If the files all have the same sampling rate, PAMGuide will attempt to concatenate them to form a single contiguous spectrogram and analysis file, which will be written to the input folder. If Write output to data to file is selected, analysis files for each individual file will also be written to a folder within the target folder. If Plot type is not set to None, plot(s) of the concatenated dataset will be produced. Plots can alternatively be produced by loading the analysis file into Viewer (see next section).
</td>
<td>
If the files all have the same sampling rate, PAMGuide will attempt to concatenate them to form a single contiguous spectrogram and analysis file, which will be written to the input folder. If outwrite=1, analysis files for each individual file will also be written to a folder within the target folder. If plottype is not set to None, plot(s) of the concatenated dataset will be produced. Plots can alternatively be produced by loading the analysis file into Viewer (see next section).
</td>
</tr>
</table>

2.8 *Viewer*

Analysis files produced by *PAMGuide* (and, in R, *Meta*) can be subsequently viewed as time series and statistical representations using *Viewer*. *PAMGuide* encodes some analysis specifications in its output files so that this metadata can be used by *Viewer*.

**MATLAB**	**R**
Select **Viewer** from the drop-down menu in the **Input** panel:<br>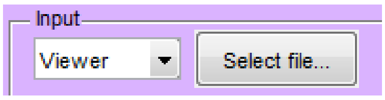<br>Select **Plot type** in the **Execute** menu (**Both**/**Time**/**Stats**/**None**).<br><br>Click **RUN**.	In R, *Viewer* is called from *Viewer.R*, contained in the *PAMGuide* folder.<br><br>Initialise *Viewer*:<br><br>`> source('Viewer.R')`<br><br>Call *Viewer*, optionally specifying **plot-type** as for *PAMGuide* (default = **Both**):<br><br>`> Viewer(plottype="Stats")`

3 Calibrating data

Calibrating data to produce absolute sound levels is essential to produce meaningful measurements of acoustic habitats for comparisons with other sites and other studies. Calibration makes it possible to quantify how much sound pressure a given level in the digital audio files corresponds to. There are three options for achieving this in *PAMGuide*:

1. Providing the end-to-end sensitivity of the entire recording system (from microphone/hydrophone to digital scale).
2. Providing the sensitivity of the recording device and the sensitivity of the microphone/hydrophone separately (a generic sensitivity for the transducer is usually given by the manufacturer).
3. Using the technical specifications of the recorder and transducer as provided by the manufacturer.

3.1 Implementation

This section describes how to input data for the above three options in *PAMGuide*; procedures to measure the parameters described are detailed in the Background section (p18).

1. End-to-end

**MATLAB**	**R**
Select **End-to-end** from the **Calib. type** drop-down menu in the **Calibration** panel:<br>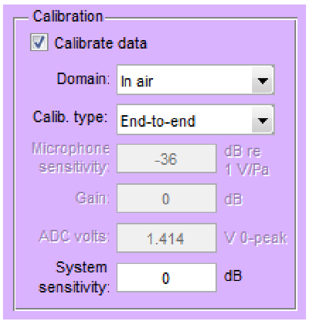	When calling *PAMGuide*, define the calibration type **ctype** as **EE** (end-to-end) and the measured sensitivity of the entire device, **Si**, in decibels, e.g.<br>`> PAMGuide(ctype="EE",Si=-159)`

Continued over...

Input measured sensitivity of entire device in the **System sensitivity** text box in decibels.	

2. Recorder + transducer

<table>
<tr><th>MATLAB</th><th>R</th></tr>
<tr><td>Select Recorder+'phone from the Calib. type drop-down menu in the Calibration panel:

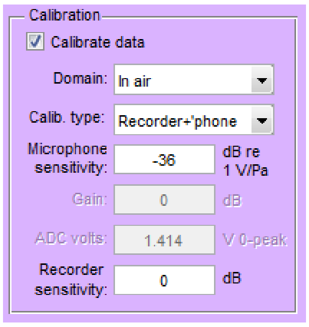

Input microphone/hydrophone sensitivity in the text box and the measured sensitivity of the recorder in the Recorder sensitivity text box in decibels.</td><td>When calling PAMGuide, define the calibration type, ctype, as RC (recorder+'phone) and the measured sensitivity of the recorder, Si, in decibels, e.g.

<code>> PAMGuide(ctype="RC",Mh=-36, Si=-20)</code></td></tr>
</table>

3. Manufacturer's specifications

MATLAB	R

Continued over...

<table>
<tr>
<td>Select Tech. specs from the Calib. type drop-down menu in the Calibration panel:

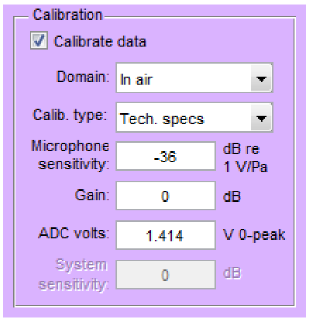

Input microphone/hydrophone sensitivity in the text box, user-defined Gain for the deployment in dB, and zero-to-peak voltage of the analogue-to-digital converter in the ADC volts text box.</td>
<td>When calling PAMGuide, define the calibration type ctype as TS (manufacturer's technical specifications), the sensitivity of the transducer, Mh, the user-defined gain settings for the deployment in decibels, G, and the zero-to-peak voltage of the analogue-to-digital converter, vADC:

<code>> PAMGuide(ctype=”MF”,Mh=-36, G=24,vADC=1.414)</code></td>
</tr>
</table>

3.2 Background

The general form of the calibration procedure converts the uncorrected signal, b, into a calibrated sound level, a, via a correction factor, S:

$$a = b - S \tag{1}$$

Here, we describe how S can be measured and/or calculated. Fig. 4 shows the signal path and conversely the calibration sequence for a typical PAM recorder, which helps to contextualise the equations presented below.

The most precise method of producing calibrated sound level measurements is to perform an end-to-end calibration of the system using a calibrated acoustic source, e.g. a reference microphone/hydrophone or a pistonphone (also known as a sound calibrator). The source is used to generate a sinusoidal sound pressure signal of known frequency, f, and zero-to-peak pressure amplitude, p_{peak}, at the system transducer (microphone or hydrophone), and this known level is then compared to the analysed signal from the system to calculate the correction factor, $S(f)$, in dB:

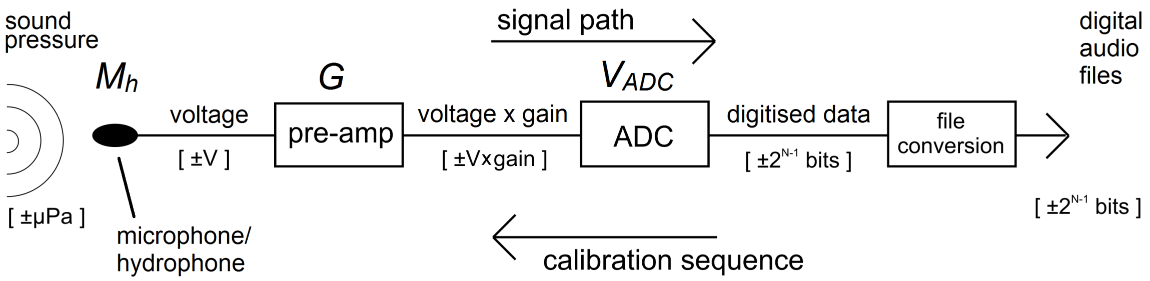

Figure 4: Signal path and calibration sequence for a typical PAM system. Values in square brackets indicate signal units at each processing stage.

$$S(f) = PP(f) + 3 - 20\log_{10}(p_{peak}) \tag{2}$$

where $PP(f)$ is the power spectrum (see Eq. 10 for details) of the digital signal, x_{bit}, at the test frequency, f, and the factor of 3 accounts for the 3 dB difference between the peak pressure amplitude, p_{peak}, and the root-mean-square (RMS) amplitude given by the power spectrum.

Alternatively, the recording system can be calibrated separately from the transducer using a signal generator. The sensitivity of the acoustic transducer is then either measured separately using a standard method (see ANSI 2005, 2012), or is taken to be the manufacturer's declared sensitivity at the test frequency, $M_h(f)$, which describes how much voltage is generated by the transducer per unit of sound pressure. To calibrate the recording system, a sinusoidal voltage of known frequency, f, and zero-to-peak voltage amplitude, V_{peak}, is applied to the transducer input of the system, and the correction factor, $S(f)$, is then

$$S(f) = PP(f) + 3 - 20\log_{10}(V_{peak}) + M_h(f) \tag{3}$$

where $M_h(f)$ has units of dB re 1 V/μPa. Note that microphone sensitivities are usually given in dB re 1 V/Pa, so a correction of -120 dB (equivalent to the 10^6 difference between Pa and μPa) is needed to convert to dB re 1 V/μPa, e.g. a microphone sensitivity of -36 dB re 1 V/Pa = -156 dB re 1 V/μPa.

Finally, the correction factor can also be computed directly from the system specifications, though this is the least reliable of the three methods described here. The specifications required are: the transducer sensitivity, $M_h(f)$, in dB re 1 V/μPa; the system gain, $G(f)$, in dB, at the frequency of interest, which describes any preamplifier gain applied to the signal; and the zero-to-peak voltage, V_{ADC}, of the analogue-to-digital converter (ADC). The correction factor, $S(f)$, is then

$$S(f) = M_h(f) + G(f) + 20\log_{10}\left(\frac{1}{V_{ADC}}\right) + 20\log_{10}(2^{N_{bit}-1}) \tag{4}$$

where N_{bit} is the bit-depth of the digital signal (e.g. 16, 24). Note that some software (e.g. MATLAB) normalises the amplitude to ± 1 when loading digital audio files, though the native format contains integers in the amplitude range $-2^{N_{bit}-1}$ to $2^{N_{bit}-1} - 1$. If such normalisation has been applied to the digital signal, the final term in Eq. 4 is omitted.

As an example of obtaining the manufacturer's specifications for Eq. 4, on p49 of the Wildlife Acoustics SM2+ user manual*, the manufacturer provides the microphone sensitivity, M_h, at 1 kHz as "Sensitivity: -36±4dB (0dB=1V/pa@1KHz)", meaning $M_h(f) = -36$ dB re 1 V/μPa at 1 kHz and within the surrounding flat frequency range of the microphone. On p47 of the manual, the ADC voltage is quoted as: "ADC: 1V rms full-scale 16-bit", meaning that $N_{bit} = 16$ and the *rms* voltage of the ADC is 1 V. V_{ADC} is the *zero-to-peak* voltage, which is given by multiplying the rms voltage by a conversion factor of $\sqrt{2}$, yielding $V_{ADC} = 1.41$ V. The system gain, $G(f)$, is defined by the user. Using these values and assuming a user-defined system gain $G(f) = 0$ dB, Eq. 4 yields $S = 51.3$ dB for this example.

While the above correction factors have been defined for specific frequencies, in practice, the frequency variation of the system sensitivity over the frequency range of the measurements may be considered sufficiently low (e.g. ± 1 dB) that a single correction factor can be applied at all frequencies within the range. Such measurements are said to be within the 'flat frequency response' of the system.

*http://www.wildlifeacoustics.com/images/documentation/SM2plus1.pdf

4 Selecting analysis parameters

PAMGuide can compute a range of acoustic metrics: broadband sound pressure level (SPL), power spectral density (PSD), 1/3-octave band levels (TOLs), and the acoustic waveform. This section describes how to define the parameters used in these analyses and how to select parameters to yield analyses with appropriate time and frequency resolutions (and file sizes) for the application. The merits of using particular acoustic metrics for specific applications are discussed in the main text (section: *Habitat characterisation*).

4.1 Analysis type

**MATLAB**	**R**
Select the desired **Analysis type** from the drop-down menu in the **Analysis options** panel:<br>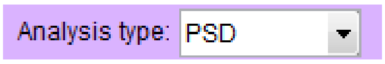<br><br>Options: **PSD**/**TOL**/**Broadband**/ **Waveform**/**TOLf**. Default = **PSD**.	When calling *PAMGuide*, define analysis type, **atype**. Options: **PSD**/**TOL**/**Broadband**/**Waveform**/ **TOLf**. Default = **PSD**. E.g.:<br>**`> PAMGuide(atype="Broadband")`**

The main difference between PSD, TOLs, and broadband noise levels is in frequency resolution. PSDs yield the sound spectrum at linearly-spaced frequency intervals, and typically offer fine-scale frequency resolution of the spectrum (depending on the window length chosen, see below). As such, they are useful for characterising fine-scale frequency variation, such as in animal vocalisations and tonal components of anthropogenic sound sources.

TOLs are spaced logarithmically in frequency, such that the frequency doubles every three 1/3-octave bands (hence 1/3-octave). Particularly at high frequencies, TOLs have lower frequency resolution than PSDs, but are efficient to compute and produce a much smaller volume of data. The logarithmic frequency spacing (constant Q) is also characteristic of the mammalian auditory system, and so TOLs or other fractional octave bands may be particularly useful for habitat characterisation (see main text). The TOLf option implements the standard 1/3-octave computation method using filter analysis (rather than based on Fourier analysis, which is faster).

The broadband level reduces the frequency content over a given range (which can be specified in *PAMGuide*, see below) to a single dB level, and so is useful when simplicity is required, though the relevant frequency range should always be reported. The broadband option also outputs the median SPL, mode SPL, and SEL for each analysis in the command line.

The pressure waveform is used to describe impulsive sounds (see main text) and sometimes animal vocalisations. As such, this analysis is typically applied to short clips of data.

4.2 Window type

**MATLAB**	**R**
Select the desired **Window type** from the drop-down menu in the **Analysis options** panel:<br>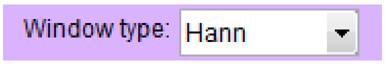<br><br>Options: **Hann/Rectangular/ Hamming/Blackman**. Default = **Hann**.	When calling *PAMGuide*, define analysis type **winname**. Options: **Hann/Rectangular/Hamming/ Blackman**. Default = **Hann**. E.g.:<br><br>`> PAMGuide(atype=”Hamming”)`

For analyses based on the discrete Fourier transform (DFT), namely the **PSD**, **TOL** and **Broadband** options (see Fig. 1), the acoustic data is divided into a series of time segments. These segments may or may not be overlapping in time (see below). It is necessary to apply a window function to these time segments to control spectral leakage, an effect whereby energy from each frequency band spreads erroneously into other frequencies. This effect is caused by the finite length of the time segments, and is an inherent feature of discrete Fourier analysis. If no window function is applied, the window is effectively a rectangular (Dirichlet) window. However, for some signals a rectangular window can introduce broad spectral leakage and correspondingly high amplitude inaccuracies, so it is generally not a good choice for monitoring of acoustic habitats, where the nature of the sound recorded is often unknown *a priori*. Instead, a 'general purpose' window such as the Hann window is advisable (Cerna and Harvey, 2000). *PAMGuide* also provides Hamming and Blackman windows as alternatives. For a detailed discussion of windows for DFT analysis, see the review by Harris (Harris, 1978). More

robust windowing techniques also exist, such as multitapering, whereby errors in spectral estimation are reduced by applying multiple windows to each segment (Thomson, 1982).

4.3 Window length

**MATLAB**	**R**
Enter the desired **Window length** in the text box in the **Analysis options** panel:<br>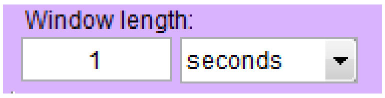<br><br>**Window length** can be input in seconds or samples (use drop-down menu to switch). If the input file is already loaded, *PAMGuide* will convert between these values an update the text box automatically.	When calling *PAMGuide*, define the window length, N, measured in samples. E.g.:<br>`> PAMGuide(N=2048)`<br>Default is the input file sample rate, Fs (= 1 second).

For PSD analysis, longer window lengths give higher frequency resolution but lower temporal resolution, and vice-versa. A compromise therefore needs to be made regarding how much time and frequency resolution is required. As a guide, if N is the frequency resolution in samples, the frequency bins are separated by Fs/N Hz, where Fs is the sampling rate in Hz. Consequently, $N > Fs$ yields frequency resolution < 1 Hz, and $N < Fs$ yields frequency resolution > 1 Hz. The default value for *PAMGuide* is $N = Fs$, yielding a frequency resolution of 1 Hz. Note that varying the window length changes the amplitude of the DFT on which the PSD is based, but the PSD is a stand measure which scales these levels to the equivalent level given by $N = Fs$.

If N is a power of 2, the DFT can be computed more rapidly, and so historically, 512- and 1024-point DFTs have been common. Such DFTs are known as Fast Fourier Transforms (FFTs), though the term is often applied to DFTs of any length. Advances in computational capabilities mean the relative inefficiency of $N \neq 2^n$ is often no longer an obstacle.

Since the **TOL** and **Broadband** analysis options in *PAMGuide* are based on the

DFT, the window length can have some effect. If the window length is too short there will not be enough frequency bins to compute the TOLs, particularly at low frequencies. For this reason, *PAMGuide* limits the window length for TOL analysis to $\geq Fs$, which yields accurate TOLs for frequencies down to 25 Hz (Mennitt and Fristrup, 2012). The precision of the frequency range for broadband analysis (see below) will also depend on the frequency resolution of the DFT. For $N < Fs$, the true low frequency limit will be the next highest bin in frequency to the input value, and the true high frequency limit will be the next lowest bin in frequency. For $N > Fs$, the input values will be accurate to 1 Hz.

4.4 Window overlap

**MATLAB**	**R**
Enter the desired **Window overlap** (as a percentage) in the text box in the **Analysis options** panel:<br>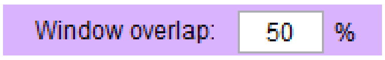 	When calling *PAMGuide*, define the window overlap, **r**, as a percentage. E.g.:<br><br>`> PAMGuide(r=75)`<br><br>Default is 50%.

Overlapping the time windows ensure that brief sounds which would otherwise be split between consecutive time segments are represented, particularly if a window function which tapers to zero at its extremities is applied (e.g. the Hann window, see Section 4.2). A 50% overlap is typically sufficient for this purpose; higher overlaps can be used to smooth spectrograms in the time domain, but do not actually improve the temporal resolution, which is determined by the length of the time segment (see previous section). Since higher overlaps produce more data points in the time domain, they also increase the volume of data produced by the analysis.

4.5 Low- and high-frequency limits

**MATLAB**	**R**
Enter the desired **Low frequency limit** (in Hz) in the text box in the **Analysis options** panel: 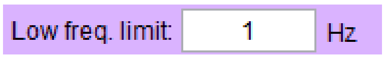 Default is 1 Hz.	When calling *PAMGuide*, define the low frequency limit, **lcut**, in Hz. E.g.: `> PAMGuide(lcut=25)` Default is Fs/N, the lowest DFT frequency bin.
Enter the desired **High frequency limit** (in Hz) in the text box in the **Analysis options** panel: 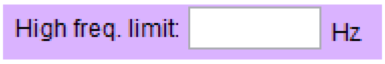 Default is the Nyquist frequency, $Fs/2$ Hz, which will appear once the input file is loaded (since the sample rate, Fs, in unknown *a priori*).	When calling *PAMGuide*, define the high frequency limit, **hcut**, in Hz. E.g.: `> PAMGuide(hcut=10000)` Default is $Fs/2$, the Nyquist limit.

Upper and lower frequency limits can be applied to limit the analysis to the frequency range over which the system sensitivity is constant (the 'flat frequency response', see Section 6), or to produce a broadband analysis over a particular frequency range of interest (e.g. in the vocal range or auditory range of a particular species). The default high-frequency limit is the Nyquist frequency, $Fs/2$, which is the highest frequency that can be resolved for a given sample rate. If high-frequency content is not of interest for a particular analysis, reducing the high frequency limit from this default will reduce the data volume of the plots and output files.

4.6 Averaging data via the Welch method

<table>
<tr><th>MATLAB</th><th>R</th></tr>
<tr><td>

Select the **Time-average data** tick box in the **Analysis options** panel:

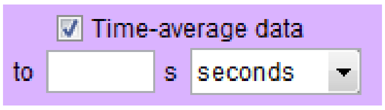

Enter the desired averaging time in seconds, or select **Factor** from the drop-down menu and enter an integer number of spectra to average.

</td><td>

When calling *PAMGuide*, define averaging factor, **welch**, as an integer. E.g.:

```
> PAMGuide(welch=10)
```

This will average 10 spectra to create each output spectrum, reducing the time resolution by a factor of 10, i.e., for 1-s windows with a 50% overlap, the time resolution becomes 5 s. Data is not lost: the data within the longer time frame is averaged via the standard Welch method (see Section 6). Default is no averaging.

</td></tr>
</table>

Time-averaging data produces more reliable measures of random signals such as sound levels in a habitat. It also saves storage and plotting time as it produces spectra with lower time resolution: this can be very significant for larger files. Time resolution can also be reduced by using longer time windows, but this does not save storage or plotting time since there is correspondingly greater frequency information (see Technical Background, p29). *PAMGuide* uses the Welch method (Welch, 1967), a standard method for time-averaging spectra.

5 Plot types

Each analysis type (PSD, TOL, Broadband, Waveform) in *PAMGuide* has pre-defined plot types for time-domain and statistical representations (see Fig. 1 in the Introduction). This section illustrates what to expect for each analysis type, for calibrated vs. uncalibrated analyses, and for time-stamped and non-time-stamped analyses.

The example data shown is 24 hours of terrestrial recording made using a Wildlife Acoustics SM2+ in Harvard Forest, Massachusetts, USA, at 44.1 kHz.

<table>
<tr><th>Time</th><th>Stats</th></tr>
<tr><td>

PSD, calibrated, time-stamped

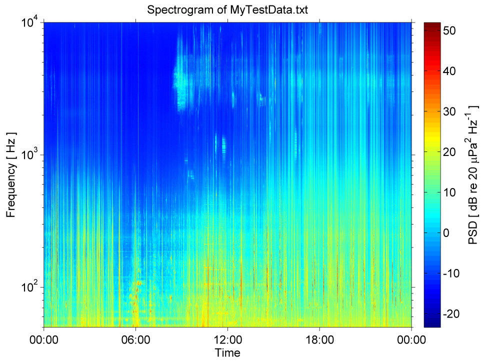

Notes: 60-s Welch averaging applied, frequency limited to 50 Hz – 10 kHz.

</td><td>

PSD, calibrated

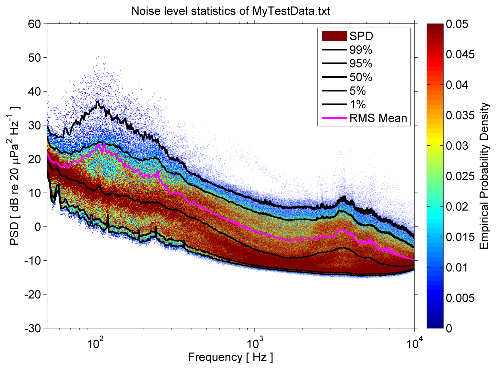

Notes: *PAMGuide* only makes SPD plots for analyses with 1000 or more data points in the time domain.

</td></tr>
<tr><td>

TOL, uncalibrated, not time-stamped

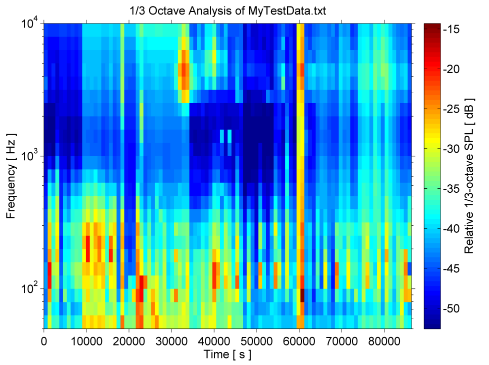

Notes: 900-s Welch averaging applied, frequency limited to 50 Hz – 10 kHz.

</td><td>

TOL, uncalibrated

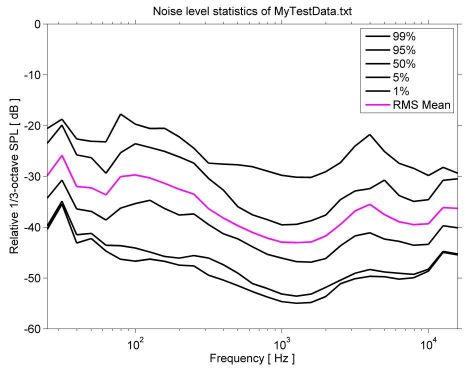

Notes: as there were less than 1000 data points in the time domain, *PAMGuide* did not plot the SPD as in the PSD example above.

</td></tr>
</table>

Continued over...

Broadband, calibrated, time-stamped	*Broadband, calibrated*
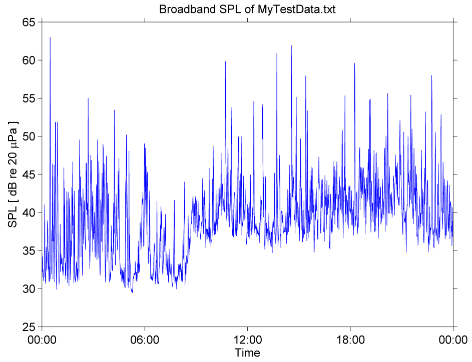 Notes: frequency range is 50 Hz — 10 kHz.	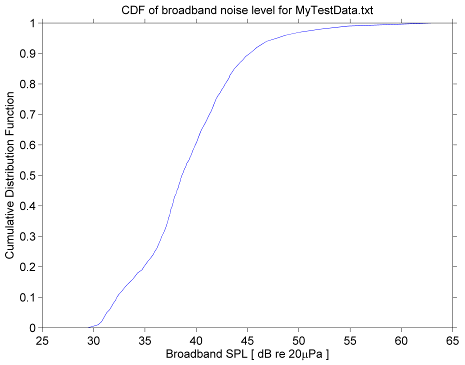 Notes: frequency range is 50 Hz — 10 kHz. The broadband option also outputs the median SPL, mode SPL, and SEL for each analysis in the command line.
Waveform, calibrated, not time-stamped	*Waveform*
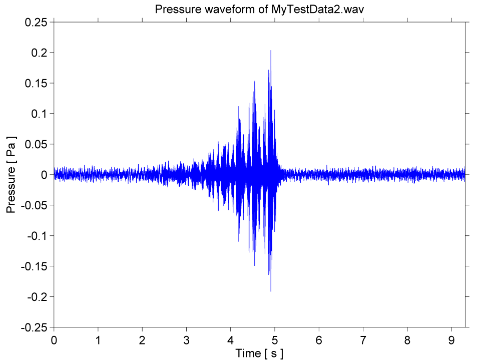 Notes: this is a short extract of a bird call recorded at the site.	Notes: there is no statistical representation of the acoustic waveform in *PAMGuide*.

PSD plots have a logarithmic frequency scale by default, which emphasises lower frequencies. A linear frequency scale can be selected by changing **Frequency scale** to **Linear** (in MATLAB) or specifying **linlog**="Lin" (in R).

6 Technical background

This section describes the signal processing steps that *PAMGuide* is based on, detailing the equations used in the code to compute acoustic metrics. Additionally, important considerations such as signal-to-noise ratio (SNR), sound exposure level (SEL), and the analysis of tonal signals are discussed. The signal processing steps to calibrate digital audio files were detailed in Section 3, above.

6.1 Computing frequency spectra

PAMGuide uses two methods to analyse the frequency characteristics of sound: discrete Fourier transforms (DFTs) are used to derive the PSD and can be used to produce 1/3-octave band levels (TOLs), while filter banks can be used to implement the standard definition of TOLs (ANSI, 2009). For the PSD, the time-domain signal, x, is transformed into the frequency domain using an N-point DFT which yields the signal energy in N linearly-spaced frequency bins between 0 and the sampling frequency, F_s. To do this, the time-series signal is divided into segments of consecutive samples, each of which is then multiplied by a window function and then transformed via the DFT. This yields a frequency spectrum for each segment. These can then be combined and plotted as a spectrogram, showing the variation in frequency content through time, or can be averaged – as will be described below – to produce a two-dimensional plot of amplitude versus frequency, forming a longer term estimate of the spectrum.

The first step in computing the PSD is to divide the time-domain signal, x, into segments, which may be overlapping in time. The mth segment is given by

$$x^m[n] = x[n + (1 - r)mN] \tag{5}$$

where N is the number of samples in each segment, $0 \leq n \leq N - 1$ (Marple, 1987), and r is the segment overlap expressed as a decimal (e.g. 0.75 for 75% overlap; see Section 4.4 for usage of overlap).

A window function is then applied to each data segment (see Section 4.2). Denoting the mth windowed data segment $x^m_{win}[n]$

$$x^{(m)}_{win}[n] = \frac{w[n]}{\alpha} x^{(m)}[n] \tag{6}$$

where w is the window function over the range $0 \leq n \leq N-1$, and α is the coherent gain factor of the window (also known as the scaling factor), which corrects for the

reduction in amplitude introduced by the window function (Cerna and Harvey, 2000).

The DFT of the mth time segment is given by

$$X^{(m)}(f) = \sum_{n=0}^{N-1} x_{win}^{(m)}[n]\text{exp}\left(\frac{-j2\pi fn}{N}\right) \quad (7)$$

where $0 \leq f \leq F_s - F_s/N$, and F_s is the sampling frequency.

The power spectrum is computed from the DFT, and corresponds to the square of the amplitude spectrum (DFT divided by N), which for the mth segment is given by

$$P^{(m)}(f) = \left|\frac{X^{(m)}(f)}{N}\right|^2 \quad (8)$$

For real sampled signals, the power spectrum is symmetrical around the Nyquist frequency, $F_s/2$, which is the highest frequency which can be measured for a given F_s. The frequencies above $F_s/2$ can therefore be discarded and the power in the remaining frequency bins are doubled, yielding the single-sided power spectrum

$$P_{SS}^{(m)}(f') = 2P^{(m)}(f') \quad (9)$$

where $0 \leq f' \leq F_s/2$. This correction ensures that the amount of energy in the power spectrum is equivalent to the amount of energy (in this case the sum of the squared pressure) in the time series. This method of scaling, known as Parseval's theorem, ensures that measurements in the frequency and time domain are comparable.

The power spectra for each of the data segments are then combined to form an array in frequency and time, PP

$$PP(f', m) = 10\text{log}_{10}\left(\frac{P_{SS}^{(m)}(f')}{p_{ref}^2}\right) - S(f') \quad (10)$$

where, for in-air measurements, p_{ref} is a reference pressure of 20 μPa, and for underwater measurements, p_{ref} is 1 μPa. The power spectrum is used to measure the amplitude of discrete frequency components (e.g. tonal vocalisations; see Section 9). However, for general characterisation of sound levels, a standardised version of the power spectrum, the PSD, is used (see below).

It is important to note that the number of samples, N, in each data segment determines number of frequency points in the DFT, and therefore the frequency

resolution of the power spectrum ($\Delta f = F_s/N$ Hz). If N is smaller, there are fewer frequency bins (number of bins is N/F_s), and each bin encompasses a wider frequency range (F_s/N Hz), meaning more spectral energy is integrated per bin. For sound with a continuous frequency distribution such as the overall sound level in a habitat, the spectral power estimates from Eq. 10 vary with N. To account for this relationship, spectra are often standardised to the levels which would result from a data segment length of F_s (i.e. one second), which yields a frequency bin width of 1 Hz. This quantity is termed the power spectral density, PSD, and is defined by:

$$\text{PSD}(f', m) = 10\log_{10}\left(\frac{1}{B\Delta f}\frac{P_{SS}^{(m)}(f')}{p_{ref}^2}\right) \tag{11}$$

with units of dB re p_{ref} μPa^2 Hz^{-1}, where $\Delta f = F_s/N$ is the width of the frequency bins, and B is the noise power bandwidth of the window function, which corrects for the energy added through spectral leakage:

$$B = \frac{1}{N}\sum_{n=0}^{N-1}\left(\frac{w[n]}{\alpha}\right)^2 \tag{12}$$

For the Hann window, B=1.5 (see Harris (1978) for other windows). The PSD gives an estimate of the power in each frequency band of 1 Hz width even if the spectral analysis has a much coarser frequency resolution (i.e., if $N < F_s$). This is appropriate for the smoothly-varying spectra frequently encountered in ambient noise monitoring and the use of a standardized 1 Hz bandwidth ensures that measurements made with different frequency resolutions are comparable. However, the scaling applied to the PSD for the purposes of standardisation mean it will not give accurate amplitude estimates for narrowband signals such as tonals. To make accurate measurement of tonal signals, the unscaled power spectrum (Eq. 11) is instead used (see Section 6.9).

For some bioacoustics applications, the absolute amplitude of the signal is often not important, and spectra are displayed with arbitrary units (relative dB, often scaled to give a 0 dB maximum, such that amplitudes have negative dB values; see Fig. 1 in main text). To produce such measurements for a particular metric, the correction factor, S, should be the maximum decibel level measured.

For long-term monitoring, averaging of spectra in frequency and/or time is frequently used to produce more manageable data. One approach is to compute PSD with the desired frequency resolution and then average these to the desired

time resolution (see Section 6.4). Alternatively, the frequency resolution can be reduced by computing TOLs (see Section 6.2).

6.2 TOLs and broadband analysis

Besides the PSD, the most common acoustic metrics for ambient noise analysis are TOLs and broadband SPL. These can be computed directly from the time-domain signal using filters, or by integrating the power spectrum, P_{SS}, over the frequency range of interest before it is converted to dB.

Historically, TOLs were computed using analogue filtering equipment, and the current standard maintains this approach using digital filters (ANSI, 2009). In the digital context, it is typically more computationally efficient to instead sum the relevant frequency bins in the power spectrum (Mennitt and Fristrup, 2012) which does not deviate appreciably from the standard except at low frequencies (difference is <1 dB for frequencies above 25 Hz; Mennitt and Fristrup 2012) which are below the range of interest for most species. In *PAMGuide*, either approach can be implemented, though only the DFT-based approach is implemented in the otherwise comparable R code (Appendix S3). The filter-based approach is specified in the relevant standard (ANSI, 2009); here, we describe the frequencies at which TOLs are assessed, and illustrate the power spectrum approach.

The 1/3-octave centre frequencies, f_c, are defined as

$$f_c = f_{ref} 10^{\frac{i-1}{10}} \tag{13}$$

where f_{ref} is a standardised reference frequency of 1 kHz, and i is an integer, such that $i \geq 1$ corresponds to $f_c \geq 1$ kHz, and $i < 1$ corresponds to $f_c < 1$ kHz (ANSI, 2009). The upper and lower bounds of each band are then given by

$$f_{upper} = f_c 10^{\frac{1}{20}} \tag{14}$$

$$f_{lower} = f_c 10^{\frac{-1}{20}} \tag{15}$$

Although the exact 1/3-octave frequencies are used for computation of noise levels, they are commonly referred to by nominal values, e.g. 1250 Hz for the band centred on 1258.9 Hz (ANSI, 2009). The SPL of the mth data segment in the 1/3-octave band centred on f_c can then be estimated by summing the outputs of the power spectrum that are within this band:

$$\text{SPL}(m, f_c) = 10\log_{10}\left(\frac{1}{p_{ref}^2}\sum_{f'=f_{lower}}^{f'=f_{upper}}\frac{P_{SS}^{(m)}(f')}{B}\right) - S(f_c) \tag{16}$$

where $P_{SS}^{(m)}(f')$ is as defined in Eq. 9.

Similarly to 1/3-octave bands, broadband SPL is the integral of acoustic energy over a given frequency range. Rather than a frequency spectrum of levels, however, broadband SPL reduces the noise level to a single number, typically over a wider frequency range than 1/3-octave bands. This simplicity is useful, but can also be problematic, since broadband SPL is often reported without indicating the frequency range covered in the analysis, confounding interpretation of the results. Its single-dimensionality can also disguise problems with system sensitivity that would be apparent in spectral representations. To ensure the validity of broadband SPL measurements, the same care should be taken in correcting for system sensitivity and gain as detailed above for PSD measurements, and the frequency range of the measurement should be given. The broadband SPL of the mth data segment (see Table 1 for units), is defined by:

$$\text{SPL}(m) = 10\log_{10}\left(\frac{1}{p_{ref}^2}\sum_{f'=f_{low}}^{f'=f_{high}}\frac{P_{SS}^{(m)}(f')}{B}\right) - S \tag{17}$$

where f_{low} and f_{high} are the lower and upper bounds of the frequency range under consideration.

6.3 Statistical metrics

Spectrograms can be averaged in time to produce two-dimensional plots of PSD vs. frequency. These are commonly used to summarise and compare noise levels at different times or locations, and to view specific frequency components with greater clarity. Though there are a number of averaging methods in use for ambient noise spectra (Merchant et al., 2012), the most common is the RMS level, where the mean is computed before it is converted to dB (i.e. the mean of P_{SS}, Eq. 9). This metric is sometimes termed 'RMS mean', 'linear-space mean', or 'arithmetic mean', and is the method used by commercial sound level meters. Here, we retain the notation used above, and define the mean of the PSD spectrogram, $\overline{\text{PSD}}$, by

$$\overline{\text{PSD}}(f') = 10\log_{10}\left(\frac{1}{p_{ref}^2}\frac{1}{M}\sum_{m=1}^{m=M}\frac{P_{SS}^{(m)}(f')}{B\Delta f}\right) - S(f') \tag{18}$$

The median level is also widely used, and is independent of whether it is computed before or after conversion to decibels. Mode levels can be computed as the maximum of the PSD (Eq. 11) probability distribution in each frequency bin. Note that these average measures can be applied to other spectral measures such as TOLs and to broadband levels in the same way.

The spectral probability density (Merchant et al., 2013) shows the distribution of noise levels based on the empirical probability density. It can be computed from the PSD as an array of normalized histograms corresponding to each frequency bin:

$$\text{SPD}(f') = \frac{1}{hM} H\left(\text{PSD}(f', m), h\right) \tag{19}$$

where $H\left(\text{PSD}(f', m), h\right)$ is the histogram of M noise levels in the PSD at frequency f', and h is the histogram bin width in units of dB re 1 μPa^2 Hz^{-1}.

6.4 Reducing time resolution

For many applications, and particularly when plotting long time-series spectrograms, the PSD may produce an unmanageable volume of data. This can be remedied by time-averaging the spectrogram to a lower time resolution. This approach is generally more computationally efficient than carrying out the original analysis with longer time segments (which does not reduce the data quantity since the frequency resolution is correspondingly higher), and is known as the Welch method (Welch, 1967). The reduced time resolution spectrogram, PSD$_W$, of a full-resolution spectrogram of (time) length M is given by

$$\text{PSD}_W(f', q) = 10\text{log}_{10} \frac{1}{p_{ref}^2} \frac{R}{M} \sum_{m=(q-1)R+1}^{m=qR} \frac{P_{SS}^{(m)}(f')}{B\Delta f} - S(f') \tag{20}$$

where M is the total number of data segments, R is the downscaling factor (e.g. R = 10 to yield 10 times fewer data segments), $Q = M/R$ is the number of averaged time segments produced, and $1 \leq q \leq Q$. Each segment of PSD$_W$ thus consists of the mean of R full-resolution segments averaged in linear space. The same process can be applied to 1/3-octave levels and broadband SPLs.

6.5 Computing the acoustic waveform

Some acoustic measurements (e.g. peak-to-peak pressure measurements of impulses; see next section) are performed on the *pressure waveform*: the time-domain acoustic pressure signal, $x_{\mu Pa}$, that was recorded by the hydrophone.

The acoustic pressure waveform, $x_{\mu Pa}$, can be computed from the digital audio signal, x_{bit}, using the calibration correction factor (see Section 3):

$$x_{\mu Pa} = \frac{x_{bit}}{10^{\frac{S}{20}}} \tag{21}$$

where S is the correction factor and x_{bit} is the digital (bit-scaled) signal, such that the amplitude range is $-2^{N_{bit}-1}$ to $2^{N_{bit}-1} - 1$.

6.6 Impulse metrics

Impulsive sounds are typically described using peak-to-peak or zero-to-peak sound pressure levels (here denoted SPL_{p-p} and SPL_{0-p}), which are computed directly from the acoustic pressure waveform, $x_{\mu Pa}$ (Eq. 4):

$$\text{SPL}_{p-p} = 10\log_{10}\left(\frac{(\max(x_{\mu Pa}) + |\min(x_{\mu Pa})|)^2}{p_{ref}^2}\right) \tag{22}$$

$$\text{SPL}_{0-p} = 10\log_{10}\left(\frac{\max(x_{\mu Pa}^2)}{p_{ref}^2}\right) \tag{23}$$

If necessary, the waveform should be cropped such that the impulse of interest has the highest amplitude in the time series. When measuring the levels of impulsive sounds, it is important to ensure that the measurements are made within the flat frequency response of the system, and to report the frequency range of the measurements so that they can be usefully interpreted.

6.7 Sound exposure level

Sound exposure level (SEL) is a cumulative measure of acoustic energy over a period of time. For impulsive sounds, it is computed from the pressure waveform, $\text{x}_{\mu Pa}$:

$$\text{SEL} = \sum_{0}^{t} \frac{x_{\mu Pa}^2}{p_{ref}^2 s} \tag{24}$$

where s is a reference time of 1 s. A time interval corresponding to the period between the occurrence of the 5th and 95th percentiles of cumulative $x^2_{\mu Pa}$ is often used (see main text, Section 4).

For continuous sound, the SEL can be computed as a summation of the power spectrum over a specified bandwidth of frequencies:

$$\text{SEL} = 10\log_{10}\left(\sum_{m=1}^{M}\sum_{f'=f_{low}}^{f_{high}}\frac{P_{SS}^{(m)}(f')/B}{p_{ref}^2 s}\right) - S = \text{SPL} + 10\log_{10}(T) \quad (25)$$

where f_{low} and f_{high} are the lower and upper bounds of the frequency bandwidth under consideration, and T is the total duration of the signal. To be meaningful and enable comparisons with other studies, SELs should be quoted with the period of time over which they were calculated.

6.8 Signal-to-noise ratio and the system noise floor

The signal-to-noise ratio (SNR) describes the difference between the amplitude of a sound and the background noise (or the system noise floor, see below). It is important to calculate the SNR since low SNR can affect the accuracy of amplitude measurements. The SNR is measured in dB and is approximated as the difference between the level of the signal plus noise and the noise level that would have been recorded in the absence of the signal. The noise level is usually approximated by the noise level immediately preceding or following the signal if it is transient of from a nearby frequency band if the signal is continuous. As a general rule, an SNR $\geq$ 10 dB yields reliable amplitude measurement.

The system noise floor is the sound level measured by a PAM device in the absence of sound. This property of PAM systems can have an important influence on the quality of sound measurements and so is important to estimate. The noise floor varies with the gain and filter settings, the sampling rate, and any processing that is performed in the device prior to storing the data and so should be estimated for each configuration. Although it may be possible to infer the noise floor from manufacturers' data, it is preferable, if possible, to measure it directly with the settings that will be used in the field. To do this, the device is set to record in a quiet location with the transducer isolated from sources of vibration. For in-air recorders, an acoustically isolated space (e.g., an anechoic chamber) is usually required for this measurement. As hydrophones are relatively insensitive in air, a quiet room will usually suffice for underwater sound recorders. The PSD of the

resulting recording can be used to determine the level of the noise floor.

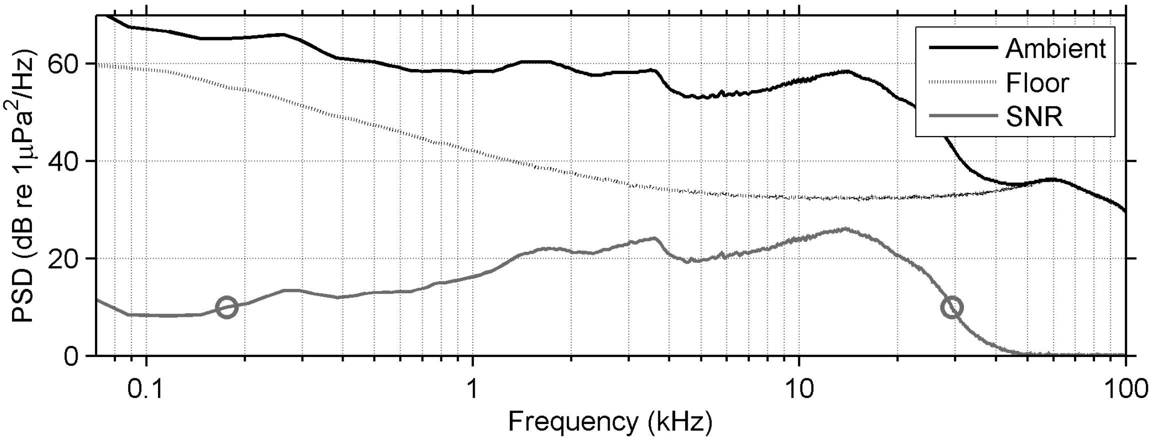

Figure 5: Power spectral density (PSD) and signal-to-noise ratio (SNR) of a 5-minute underwater noise recording in deep water off the Azores using a 8192-point FFT. Sampling rate: 240 kHz. 'Ambient' denotes the ambient noise signal (which contained vessel sounds and sperm whale clicks); 'Floor' is the system noise floor; 'SNR' is the difference between these in decibels. The ambient noise levels are only reliable where the SNR $\geq$ 10 dB, between 200 Hz and 29 kHz (marked by circles).

The system noise is one factor determining the suitability of a PAM device to make measurements in a given environment. A rule of thumb is that the system noise should be at least 10 dB below the signals of interest in each frequency band (e.g., each bin of the PSD) for spectral measurements to be reliable. For habitat monitoring applications, this implies that the system noise should be 10 dB lower than the lowest anticipated ambient noise at all frequencies of interest. This can be especially challenging to achieve at high frequencies, and most aquatic sound recorders will be system noise-limited at frequencies above about 20 kHz (Johnson, Partan, and Hurst, 2013). Because of this, the signal-to-noise ratio (SNR), i.e., the decibel difference between the signal and the noise floor, is a key metric to assess data quality that should be reported for all sound measurements. It is good practice to show the system noise floor or SNR on plots of ambient noise spectra (e.g., Fig. 5) to demonstrate the frequency range over which the system noise has little impact on the recorded levels. Whether the system noise floor has constrained the ability to record sound levels is often evident in plots of the spectral probability density (Merchant et al., 2013), wherein a sharp cut-off in sound levels recorded may be observed (see Fig. 3, main text).

6.9 Tonal signals

Unlike measurements of the overall sound level in a habitat, the amplitudes of discrete tonal signals (such as tonal vocalisations, blade-passing tones in ship or windmill noise, or sinusoidal tones used for equipment calibration) are measured either from the filtered time series or using the unscaled power spectrum (Eq. 10). If the power spectrum length, N, is chosen so that the tone frequency falls within a single frequency bin, the power spectrum level in that bin will represent the power amplitude of the plus any noise present in that bin. If the SNR within the bin is greater than 10 dB, the power spectrum level is a close estimate of the tonal power and the noise contribution can be neglected. If the tone frequency falls near the edge of a DFT band, it will contribute to the power spectrum level in more than one bin, and the power in both bins must be summed (before conversion to dB) to estimate the overall power of the tone.

7 *PAMGuide* index

This section provides an exhaustive list of *PAMGuide* parameters and their relevant page references in the tutorial.

7.1 MATLAB

Panel	Parameter	Page
**Input**	One file	5
	Batch	13
	Viewer	15
**Analysis options**	Analysis type	21
	Window type	22
	Window length	23
	Window overlap	24
	Low freq. limit	25
	High freq. limit	25
	Reduce time resolution	26
	Load file in stages	13
**Time stamp**	Time stamp data	11
**Calibration**	Calibrate data	7, 16
	Domain	8
	Calib. type	16
	′phone sensitivity	16
	Gain	16
	ADC voltage	16
	Recorder sensitivity	16
**Execute**	Plot type	6, 27
	Frequency scale	28
	Write output data to file	7

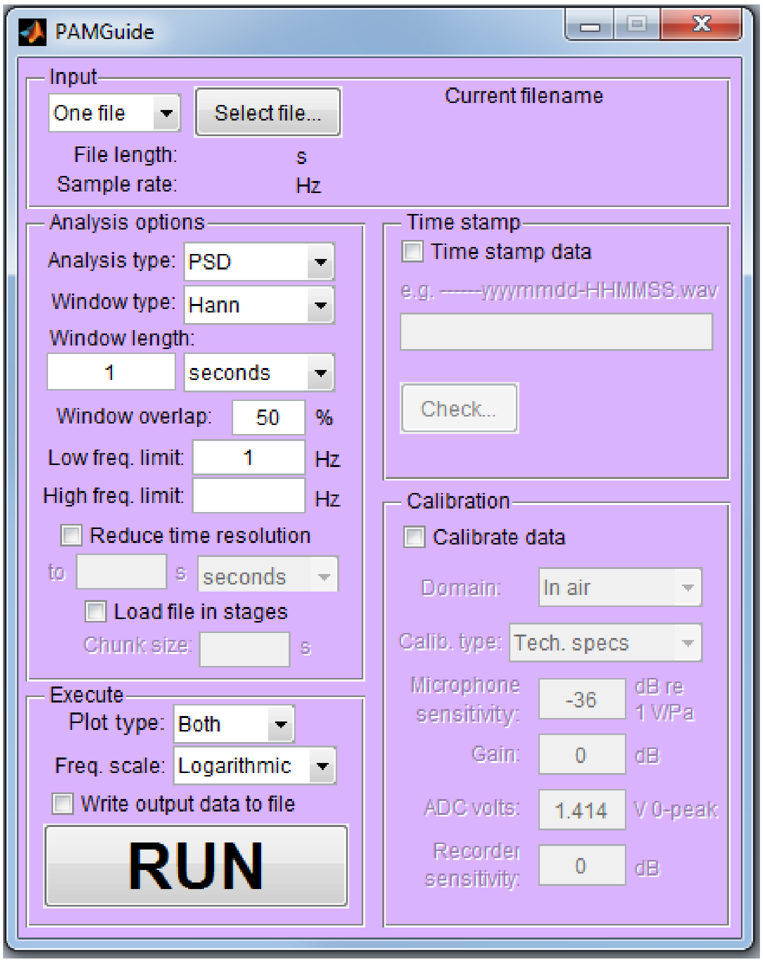

Figure 6: *PAMGuide* graphical user interface in MATLAB.

7.2 R

**Parameter**	**Default**, options	**Page**
atype	**"PSD"**, "TOL", "Broadband", "Waveform"	21
plottype	**"Both"**, "Time", "Stats", "None"	6, 27
envi	**"Air"**, "Wat"	8
calib	**0**, 1	7, 16
ctype	**"TS"**, "EE", "RC"	16
Si	-159	16
Mh	-36	16
G	0	16
vADC	1.414	16
r	50	24
N	Fs	23
winname	**"Hann"**, "Rectangular", "Hamming", "Blackmann"	22
lcut	Fs/N	25
hcut	$Fs/2$	25
timestring	""	11
outwrite	**0**, 1	7
welch	""	26
chunksize	""	13
linlog	**"Log"**, "Lin"	28

References

ANSI (2005) ANSI/ASA S1.15-2005/Part 2 (R2010) Measurement Microphones — Part 2: Primary Method for Pressure Calibration of Laboratory Standard Microphones by the Reciprocity Technique. American National Standards Institute.

ANSI (2009) ANSI/ASA S1.11-2004 (R2009) Specification For Octave-band And Fractional-octave-band Analog And Digital Filters. American National Standards Institute.

ANSI (2012) ANSI/ASA S1.20-2012 Procedures for Calibration of Underwater: Electroacoustic Transducers. American National Standards Institute.

Cerna, M. & Harvey, A.F. (2000) The fundamentals of FFT-based signal analysis and measurements, Application Note 041. Tech. rep.

Harris, F.J. (1978) On the use of windows for harmonic analysis with the discrete Fourier transform. *Proceedings of the IEEE* **66**, 51–83.

Johnson, M., Partan, J. & Hurst, T. (2013) Low complexity lossless compression of underwater sound recordings. *The Journal of the Acoustical Society of America* **133**, 1387–1398.

Marple, S.L. (1987) *Digital Spectral Analysis with Applications*. Prentice Hall Inc, NJ.

Mennitt, D.J. & Fristrup, K.M. (2012) Obtaining calibrated sound pressure levels from consumer digital audio recorders. *Applied Acoustics* **73**, 1138–1145.

Merchant, N.D., Barton, T.R., Thompson, P.M., Pirotta, E., Dakin, D.T. & Dorocicz, J. (2013) Spectral probability density as a tool for ambient noise analysis. *The Journal of the Acoustical Society of America* **133**, EL262–EL267.

Merchant, N.D., Blondel, P., Dakin, D.T. & Dorocicz, J. (2012) Averaging underwater noise levels for environmental assessment of shipping. *The Journal of the Acoustical Society of America* **132**, EL343–EL349.

Thomson, D.J. (1982) Spectrum estimation and harmonic analysis. *Proceedings of the IEEE* **70**, 1055–1096.

Welch, P. (1967) The use of fast Fourier transform for the estimation of power spectra: A method based on time averaging over short, modified periodograms. *IEEE Transactions on Audio and Electroacoustics* **15**, 70–73.
